# Dynamics of organic matter in algal blooms on the Greenland ice sheet

**DOI:** 10.1038/s41598-025-92182-7

**Published:** 2025-03-10

**Authors:** Pamela E. Rossel, Runa Antony, Rey Mourot, Thorsten Dittmar, Alexandre M. Anesio, Martyn Tranter, Liane G. Benning

**Affiliations:** 1 Interface Geochemistry Section, GFZ Helmoltz Centre for Geosciences, Potsdam, Germany; 2 National Centre for Polar and Ocean Research, Ministry of Earth Sciences, Vasco da Gama, Goa India; 3 Aix Marseille Univ, Université de Toulon, CNRS, IRD, MIO, Marseille, France; 4 Institute for Chemistry and Biology of the Marine Environment (ICBM), Carl von Ossietzky University Oldenburg, Oldenburg, Germany; 5 Helmholtz Institute for Functional Marine Biodiversity (HIFMB) , Oldenburg, Germany; 6 Department of Environmental Science, Aarhus University, Frederiksborgvej 399, 4000 Roskilde, Denmark; 7 Department of Earth Sciences, Freie Universität Berlin, 12249 Berlin, Germany

**Keywords:** Snow and glacial ice algal blooms, Dissolved and particulate organic matter, Carbon dynamics, Fourier-transform ion cyclotron resonance mass spectrometry, Arctic, Greenland ice sheet, Cryospheric science, Biogeochemistry, Carbon cycle

## Abstract

Surface melting supports the development of pigmented algal blooms on the Greenland Ice Sheet, decreasing albedo and further accelerating melting. The interplay between carbon-fixing algae and carbon-respiring heterotrophic microorganisms ultimately controls the amount and composition of organic matter (OM) and thus the ice and snow color. Yet, the dynamics of microbially-derived OM on the Greenland Ice Sheet remain unclear. To address this knowledge gap, we incubated in situ algae-dominated snow and ice samples under light and dark conditions and characterized the changes in dissolved and particulate OM (DOM and POM) with the help of ultrahigh-resolution Fourier transform ion cyclotron resonance mass spectrometry. We show that glacier ice-algae habitats are dominated by highly unsaturated and aromatic compounds resistant to bio- and photo-degradation. In contrary, snow-algae habitats are enriched in bioavailable and more photosensitive unsaturated aliphatics and sulfur- and phosphorus-containing compounds. In both habitats, light exposure increased water-soluble DOM compounds derived from POM, which accounted for ~ 50–70% of the initial DOM composition. Of the initial DOM, 35–50% were heterotrophically degraded in the dark, while light alone photodegraded 6–16%. The significant accumulation of light-absorbing aromatics from POM and DOM at the end of the ice-algae experiments, underscore the greater impact of glacier ice-algae habitats on altering glacier color and accelerating melting.

**Supplementary Information:**

The online version contains supplementary material available at 10.1038/s41598-025-92182-7.

## Introduction

Glaciers and ice sheets are Earth’s second largest freshwater reservoir^1^ and cover roughly 10% of our continents. Global warming has increased melting^2,3^, enhancing the habitability of snow and ice surfaces by microalgae^4^. Pigmented snow- and glacial ice-algal blooms increase the absorption of solar radiation, decrease albedo, and magnify surface melt^5–7^. In these biomes the interactions between carbon-fixing algae^7^ and heterotrophic microorganisms regulate the dynamics and composition of organic matter (OM), which in turn affect pigmented algal blooms development and surface color. On the Greenland Ice Sheet (GrIS), supraglacial biomes can be both source and sink of dissolved and particulate OM (DOM and POM)^8,9^. Pigmented algae exposed to 24 h summer light, generate bioavailable DOM for heterotrophic communities^10,11^. A fraction of this DOM can be altered during transit through the drainage system^12^, while another fraction escapes degradation^8^ affecting downstream ecosystems^8,13–15^. However, the concentrations, compositions and dynamics of DOM/POM pools in supraglacial systems are poorly constrained. We do not know what role 24 h light exposure or heterotrophic processes play in shaping the OM molecular makeup in algal blooms and thus in the degradation or accumulation of OM with light-absorbing properties. Also, it is unclear what changes in OM pools arise during bloom development. These knowledge gaps limit our ability to predict how pigmented algae blooms govern GrIS darkening^5,7,16–18^ and glacier OM export today and in the future^8,14,15,19,20^.

To address this, we assessed the influence of light vs. darkness on OM dynamics of red snow-algal and purple glacier ice-algal blooms in long (24 days) in situ incubations. We simulated GrIS conditions (Methods and Supplementary Figs. S1 and S2) and followed the effects that net autotrophic and heterotrophic processes have on DOM/POM composition by untargeted molecular analysis via ultrahigh resolution Fourier transform ion cyclotron resonance mass spectrometry (FTICR-MS, see Methods)^21^. We tested if: (1) OM composition of the blooms is linked to the dominating algae type, (2) solar radiation stimulates autotrophic photoproduction of DOM and abiotic release of particle-bound OM and (3) heterotrophy differentially alters OM composition in the two contrasting algal habitats, perturbing the rate of darkening due to the accumulation of light-absorbing refractory aromatics.

## Results and discussion

### Algal diversity, DOC concentrations and OM molecular characteristics

**Fig. 1:**
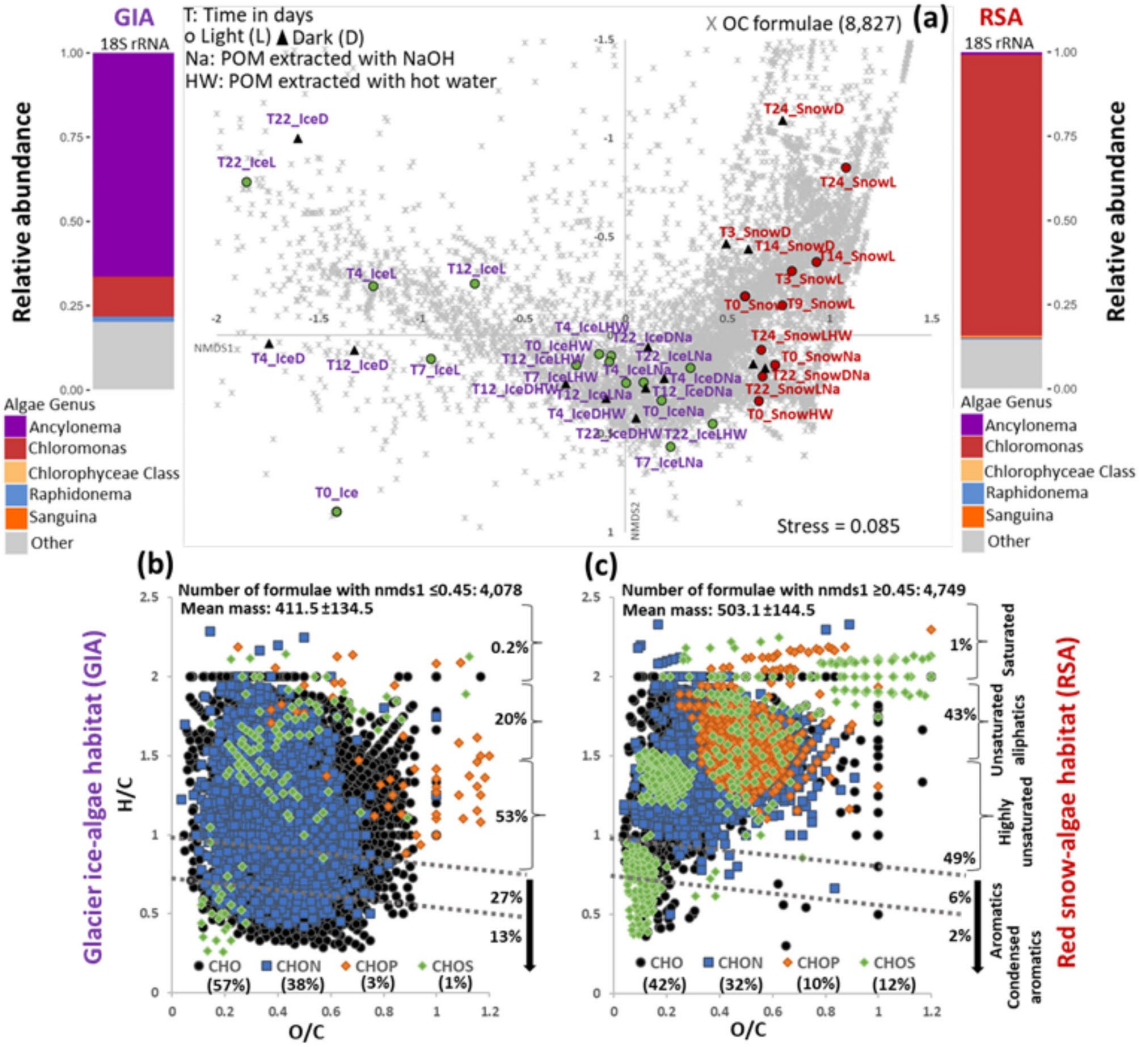
Compositional differences in all OM (both DOM and POM and both light and dark samples) in the purple glacier ice-algae (GIA) and red snow-algae (RSA) dominated habitats based on Bray Curtis dissimilarity. (**a**) Non-metric multidimensional scaling (NMDS) of ice-algae (purple labels at left) and red snow-algae (red labels at right) samples, with the vertical and horizontal axes (NMDS1 and NMDS2) explaining the OM molecular variability (grey x); the legend in the upper left details the information in the sample labels: type of incubation (dark, D and light, L) and time (T in days) for DOM and POM samples, and type of extracts for POM (NaOH and hot water). Plotted at left and right of the NMDS plot in (**a**), is the relative abundance of algal species in the initial samples (T0_Ice and T0_Snow) based on 18S rRNA sequencing analyses (see further information in methods). (**b**) and (**c**) van Krevelen diagrams with molecular formulae plotted according to their H/C and O/C ratios using NMDS1 loadings ≤ 0.45 for ice-algae (at left) and ≥ 0.45 for snow-algae (at right); displayed in each diagram is the overall contribution (in %) of saturated, unsaturated aliphatics, highly unsaturated, aromatics and condensed aromatics (Tables S4 and S5) as well as the elemental composition of the formulae (CHO, CHON, CHOS, CHOP) all relative to the total number of formulae in each habitat signal (4,078 in GIA and 4,749 in the RSA). Note: due to insufficient particulate material in the red snow at intermediate time steps, POM could only be analyzed in the initial and final time point sample.

18S rRNA marker gene sequencing confirmed that the snow-algae *Chloromonas* and the glacier ice-algae *Ancylonema*^7,17,22^ dominated the eukaryotic community composition in each habitat (90% and 65% respectively, Fig. 1). Snow samples contained solely snow-algae taxa, whereas ice samples still contained snow-algae remnants (12%, Fig. 1) from snow-melt, as previously reported^4^. The two habitats differed not only in algae taxonomy, but also in algae life cycle stage, with snow-algae normally considered to be close to the resting stage and ice-algae still actively growing and dividing^23^.

Over the 24-day incubations, dissolved organic carbon (DOC) concentrations (0.7 ± 0.05 in snow and 1.2 ± 0.1 mg-C l^-1^ in ice) increased ca. 2- to 6-fold (Fig. S3). Water-soluble (POM-HW) and water-insoluble extracts (POM-NaOH, Fig. S1) also differed with water solubilizing 2-fold less DOC from snow compared to ice (1184 ± 56 vs. 2128 ± 216 mg-C l^-1^), while water-insoluble DOC concentrations were similar (885 ± 57 vs. 1090 ± 187 mg-C l^-1^, Fig. S3). In the POM-extracts of snow, DOC concentrations remained similar over time, while in the ice they decreased 1 to 2.5-fold (Fig. S3). The different algal genera also produced compositionally different OM (Figs. 1 and S4, Tables S1, S2 and S3). Snow-algae had significantly more phosphorus and sulfur compounds compared to ice-algae (Figs. 1b-c and S5, Tables S4 and S5). Up to 1% of the phosphorus- and sulfur-containing compounds in the initial snow-algae POM were also present in the initial ice-algae POM, likely due to the snow-algae present in the initial ice-algae sample (Table S1).

Nitrogen-containing compounds made up 30–38% of the total formulae in both habitats, but in snow, they were 2-fold higher in DOM, whereas in ice, they were 13-fold higher in POM (Figs. 1b–c and S4). Differences in abundance of phosphorus- and nitrogen-containing compounds between these habitats likely reflect algae metabolic activity^24^ and nutrient availability^25^. In snow, nutrients are from dry/wet deposition, while on glacial ice, nutrients are from snow/ice melting and mineral dissolution^1,25^. Like other algae^26^, snow- and ice-algae may use diverse strategies for phosphorus uptake. These adaptations, along with the difference in nutrients, and algae life cycle stages, are likely responsible for the higher abundance of phosphorus compounds in snow compared to ice-algae OM. In algae, sulfur is essential for metabolism and environmental adaptations^27^ and in our snow, sulfur compounds likely reflect *Chloromonas* response to oxidative stress from high solar radiation^28^. In contrary, glacier ice-algae, with their darker purple pigments^29^, are likely better adapted to light stress and may not actively produce sulfur compounds.

Unsaturated aliphatics (H/C ratios > 1.5), which are linked to labile algae-derived OM^30^, were 2.5-fold more prevalent in snow than ice. In ice, almost all unsaturated aliphatics were POM-derived (Figs. 1b–c, S4 and S6, Tables S4 and S5). In such a bloom, POM is mainly algal (Supplementary note 1), as confirmed by protein analyses on same sample types^31^. Thus some labile unsaturated aliphatics may include water-soluble lipids and proteins from algae^6,8,32^. However, the advantage for snow-algae of releasing unsaturated aliphatics when they transition to the cyst life stage is unclear (Fig. S4d). Highly unsaturated, aromatics and condensed aromatics (H/C ratios < 1.5), were especially higher in the ice-algae samples. This aligns with the DOM composition reported from a similar glacier ice-algal habitat^12^ but is distinct from snow-algae habitat (Figs. 1 and S6, Tables S4 and S5).

Condensed aromatics were significantly more abundant in ice-algae (13%) compared to snow-algae OM (2%) and in snow, almost all condensed aromatics were in DOM (Figs. 1, S4 and S6). The origin of condensed aromatics on glaciers is unclear and could be allochthonous^19^. If formed from incomplete combustion of biomass or fossil fuel burning, they represent particulate wind-delivered black carbon (BC) known to reduce snow albedo^33^. However, if all our condensed aromatics would be BC or dissolved BC^15,19^, it is unclear why BC would be more abundant in ice- than snow-algae blooms. Alternatively, our condensed aromatics could be algae-produced compounds. Indeed many aromatics and condensed aromatics in ice (66% and 72%, Table S4) and snow (95% and 96%, Table S5) could belong to the extended molecular category of oxy-aromatic phytochemicals, considered abundant in plants^34^. Some of these compounds might include phenolic to polyphenolic metabolites produced by algae^35^, falling within the mass range of those in our samples (100–800 Da, Table S4 and S5).

Among phenolic metabolites, sugar-containing tannins and flavonoids, can assist in UV protection, pigmentation, cell-signaling and antioxidant activity^35,36^. In glacier ice-algae, the abundant purpurogallin phenolic pigments aid in photoprotection against excessive solar radiation and cellular heat generation^18,29^. In snow-algae, aromatics were mainly present in DOM and had lower O/C ratios and higher sulfur contents (Figs. 1c and S4d, Tables S4 and S5). Since glacier ice-algae phenolic pigments were previously documented only in higher plants, the presence of sulfur-free and sulfur-containing aromatics in ice- and snow-algae, respectively, may also be related to their taxonomy (ice-algae are Zygnematophytes and snow-algae are Chlorophytes). Aromatics, with their conjugated carbon double bonds, absorb light, crucially influencing the light-absorption and optical properties of chromophoric DOM (CDOM)^21^, with CDOM in these blooms closely linked to algae biomass and pigments^37^. We demonstrate that these blooms produced distinctive OM patterns, containing aromatic compounds, particularly abundant in glacier ice-algae habitats (Figs. 1 and S4), that will invariably influence the GrIS color. Their impact on GrIS darkening depends not only on their light-absorbing properties, but also on their potential accumulation over the summer, influenced by their production and degradation. To constrain OM production and degradation, we assessed molecular variations over time under light and dark conditions (Fig. 1b–c, Tables S4 and S5).

### Release of OM from particles in light

**Fig. 2:**
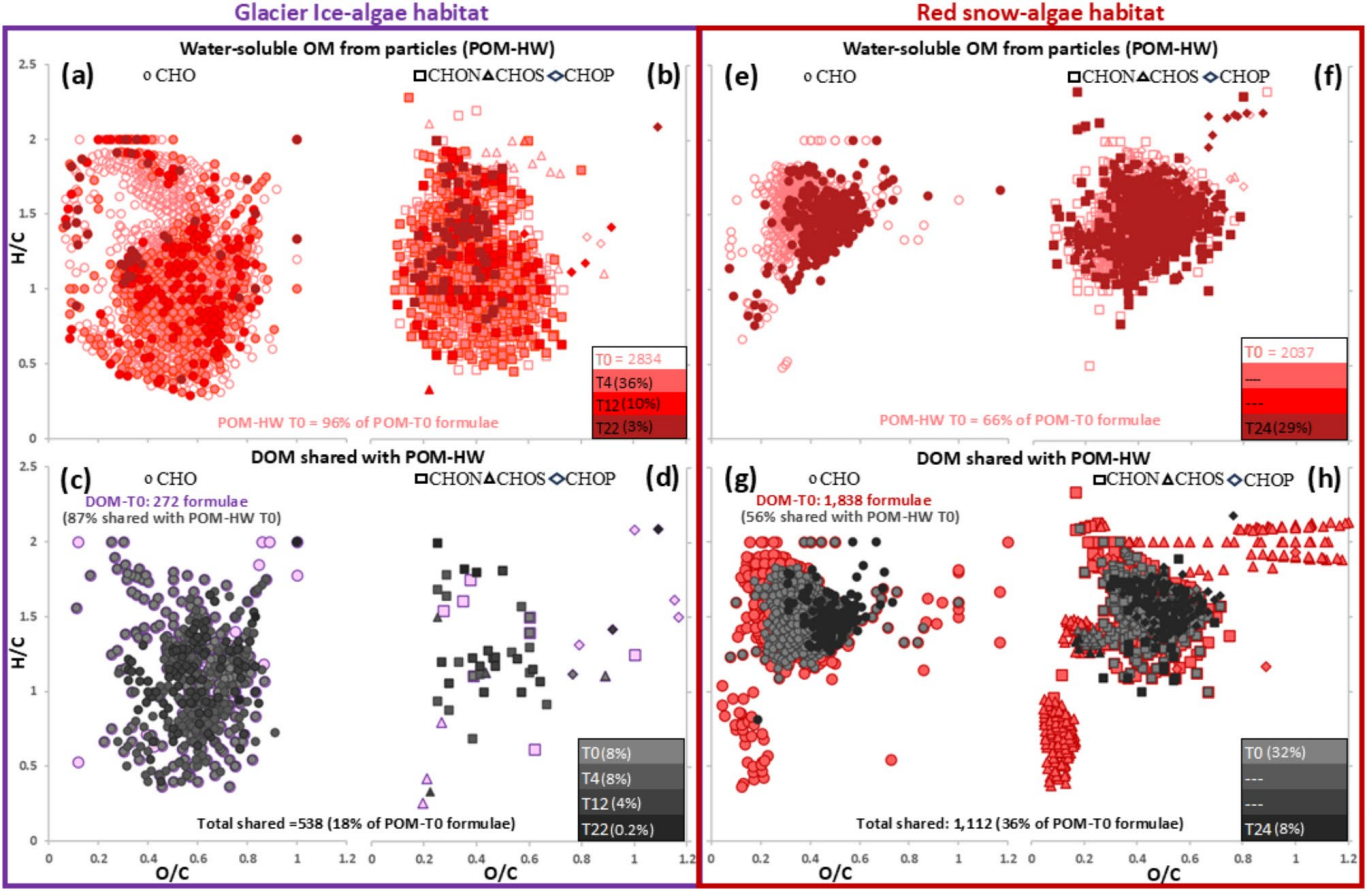
Time resolved influence of solar radiation on the transfer of water-soluble OM from POM to DOM in the glacier ice-algae (left panel) and red snow-algae (right panel) experiments. van Krevelen diagrams with molecular formulae according to their H/C and O/C ratios for (**a**) and (**b**) water-soluble ice-algae OM at T0 (empty) and over time (filled red symbols) only under light conditions; (**c**) and (**d**) ice-algae DOM at T0 (purple) and DOM shared with water-soluble ice-algae OM over time (dark tone symbols); (**e**) and (**f**) water-soluble snow-algae OM at T0 (empty) and over time (filled red symbols) only under light conditions and (**g**) and (**h**) snow-algae DOM at T0 (red) and DOM shared with water-soluble snow-algae OM over time (dark tone symbols). Symbol shapes indicate organic compounds without (CHO) or with heteroatoms (CHON, CHOS, and CHOP). The contribution of formulae over time, indicated by the increasing dark color intensity in each panel, is expressed as percent relative to the total number of formulae in POM-T0 for glacier ice-algae (total 2,945 formulae) and red snow-algae experiments (total 3,066 formulae); for details of POM-T0 for each experiment see Tables S4 and S5. For ease of viewing DOM-T0 symbols in ice and snow are displayed in purple and red one size bigger and those uncover by dark symbols represent formulae in DOM-T0 that were not shared with the initial POM. Note: due to insufficient particulate material in the red snow at intermediate time steps, POM could only be analyzed in the initial and final time point sample.

The light exposure induced DOM production or release from POM can occur abiotically or biotically through photosynthesis. Photoproduced algal DOM may dominate over heterotrophy and progressively increase, especially during the early experimental period when photosynthesis was not DIC limited. We evaluated biotic and abiotic changes by following the release of water-soluble and water-insoluble compounds from POM (Fig. S1), as evidenced by the shared formulae between POM and DOM found exclusively in light incubated samples. We show that up to 18% and 36% of the initial ice- and snow-POM formulae were transferred to their DOM pools (Figs. 2 and S7, Tables S4 and S5). Despite constituting less than half of the initial POM, these formulae accounted for > 50% and 70% of the DOM-T0 composition (Figs. 2 and S7).

Autotrophically produced DOM was particularly evident during the first nine days of the experiments, with photoproduced formulae comprising ~ 50% or more than those in DOM-T0 (Fig. S8). Such a DOM photoproduction has been also documented in Antarctic clean snow^38^. In both algal experiments, the autotrophically produced formulae were 29–41% of those released from POM (Fig. S9, Tables S4 and S5), suggesting that light alone could induce additional processes that refill the DOM pool, potentially explaining more than half of its composition (Fig. S9). Over time, water-soluble formulae decreased, along with those transferred to DOM in the ice samples (Fig. 2a–d), while water-insoluble displayed more variability (Fig. S7a-d). This trend was mirrored in the DOC from DOM and POM extracts (Fig. S3). Such a DOC increase could indicate abiotic leaching, a process inferred for cryoconite particulates^39^. This may be more common, yet not well studied POM-DOM transfer mechanism in algal-rich ice/snow environments. In snow, water-soluble and -insoluble formulae decreased with time, alongside with those transferred (Figs. 2e–h and S7e-h). However, unlike in ice, these changes were not reflected in the DOC (Fig. S3).

In the ice experiments, the transferred OM was related to unsaturated aliphatics and oxygen-rich highly unsaturated (O/C > 0.5) and aromatics (Fig. 2c–d, Table 1). Despite the fact that ice-algae POM had aliphatics and highly unsaturated nitrogen-containing compounds, < 4% of those in POM-T0 were transferred or were rapidly used up and thus almost absent in DOM (Fig. 2b,d). In snow, transferred OM contained nitrogen and phosphorus, and was slightly oxygen-poorer, and related to highly unsaturated and unsaturated aliphatics (Figs. 2g–h and S7g-h, Table 1). Overall, photoproduction diversified the DOM-T0 composition in both habitats, increasing the number of formulae without heteroatoms (i.e., CHO) in the ice vs. nitrogen and sulfur compounds in the snow. Some of these sulfur compounds were aromatic and present only in the snow-DOM likely due to autotrophic photoproduction (Figs. 2g–h and S8c-d). Our findings indicate that bloom-derived DOM composition depends on autotrophic metabolic production and the overall POM composition. While POM is algae dominated (Supplementary Note1), it could also contain a minor OM fraction from other microorganisms or atmospheric deposition, that can affect the DOM pool by releasing OM during light exposure, contributing to the DOC released from glaciers^14,40^. The role of algal blooms is however, relevant since their DOC concentrations can be over one-fold higher (Fig. S3) than in ice/snow surfaces without blooms (usually < 0.5 mg-C l^-1^)^15,19,20^. The bloom-derived DOM will have a greater impact on GrIS surface darkening, as during the diurnal freeze-thaw cycles this OM will become ice-locked. Although part of the ice sheet surface DOM is exported^9,14^, the daily freeze-in, the slow DOM circulation on the weathering crust^12^ and the presence of “sticky” exopolymeric substances in these habitats^41^, likely maintain the DOM composition and prevent its degradation thus fostering its accumulation over time. We found higher DOC and aromaticity in the ice vs. the snow samples. However, aromatics in DOM are easily photodegraded, which supplies labile DOM for heterotrophic microorganisms^42^ and thus likely remove aromatics at the end of the summer.

### Alteration of DOM

**Fig. 3:**
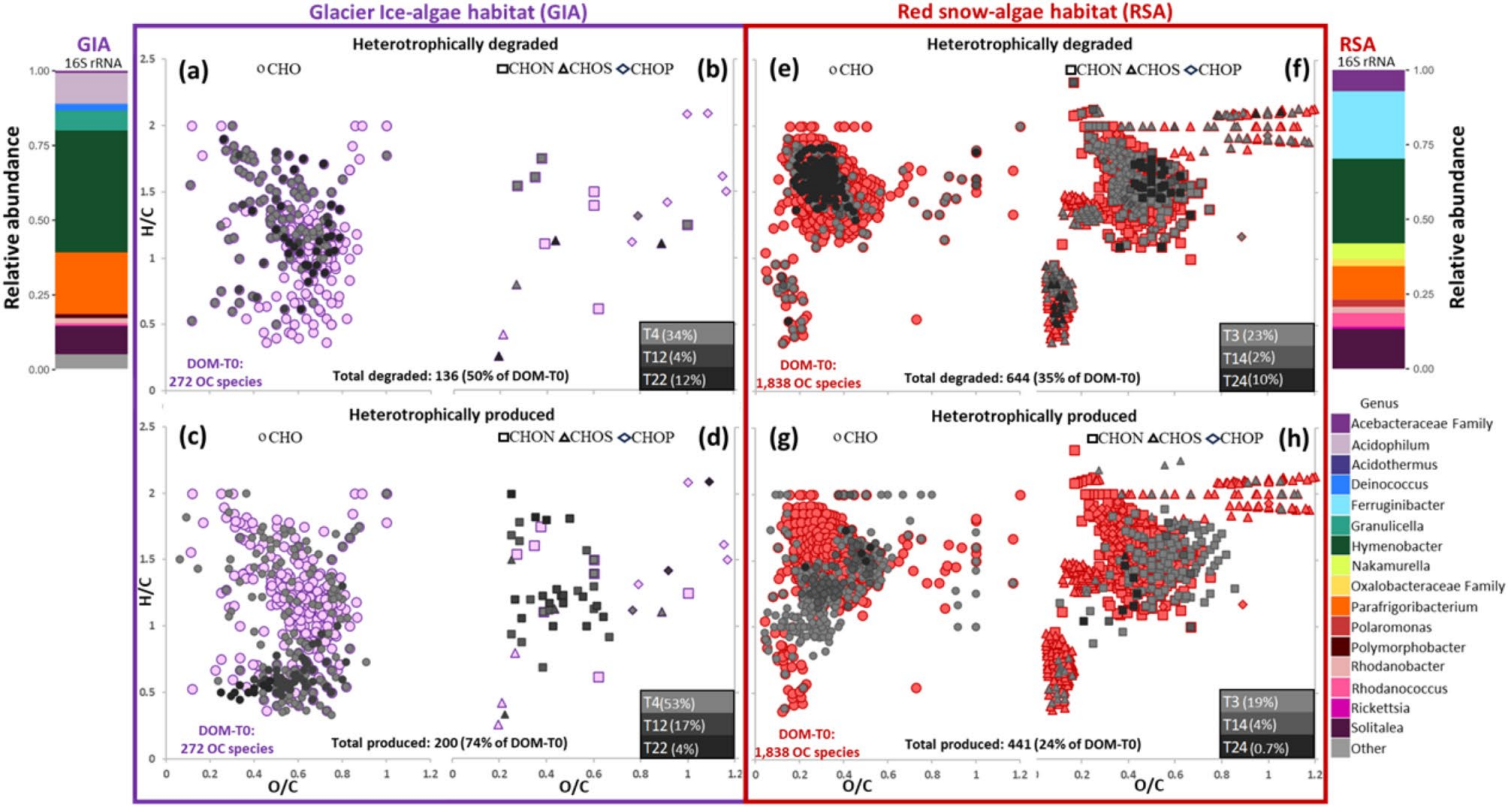
Heterotrophic molecular signals in DOM of glacier ice-algae (GIA) and red snow-algae (RSA) dominated habitats at dark conditions. van Krevelen diagrams with molecular formulae according to their H/C and O/C ratios for (**a**) and (**b**) initial DOM (DOM-T0, purple) and progressively degraded or (**c**) and (**d**) produced formulae over time due to heterotrophy in glacier ice-algae experiments (dark tones); (**e**) and (**f**) initial DOM (DOM-T0, dark red) and progressively degraded or (**g**) and (**h**) produced formulae over time due to heterotrophy in red snow-algae experiments (dark tones). Heterotrophic degradation was related to a consistent decrease in the mass peak intensity of molecular formulae until they either reached zero by the specified time or they decreased consistently without reaching zero at the end of the experiment. Heterotrophic production of molecular formulae was related to a consistent increase in mass peak intensity until they reached a maximum by the specified time. Symbol shapes indicate organic compounds without (CHO) or with heteroatoms (CHON, CHOS, and CHOP). Both degradation and production (dark tones) expressed gradual change over time in percentage relative to the total number of formulae in DOM-T0 in glacier ice-algae or red snow-algae (for details see Tables S4 and S5). For ease of viewing symbols for the DOM-T0 in ice and snow habitats are displayed in purple and dark red one size bigger than those produced or degraded. Plotted at left and right of figures (**a**) and (**f**) is the relative abundance of microbial species in the initial samples (T0_Ice and T0_Snow) based on 16S rRNA sequencing analyses (see further information in methods).

DOM changes in the dark samples revealed that ~ 50% (ice) and 35% (snow) of the DOM-T0 formulae, were heterotrophically degraded (Figs. 3a–b and e–f), aligning with the high bioavailability and rapid turnover of glacier DOM^14,43^. This heterotrophically degraded DOM was more diverse compared to the photodegraded DOM that represented a far smaller fraction (6–16% were solely degraded in light-incubated samples; Fig. S10). This contrasts with ~ 70% of the DOM formulae photodegraded in bacterial-dominated clean snow^38^. Our samples were exposed to light radiations high enough to breakdown photo-reactive DOM^44^, although the experimental bottles filtered out the highest energy of UV-B (see Methods), and thus the magnitude of the photochemical effects are more conservative. Our results suggest that our microalgae-DOM was less photosensitive (Fig. S10) likely due to the algae producing fresher, less aromatic DOM^30^. Alternatively, glacier microalgae, which are physiologically adapted to strong solar radiation^29^, may produce photoresistant DOM as reflected especially in our ice experiments (Fig. S10). In these, highly unsaturated and unsaturated aliphatics without heteroatoms were heterotrophically degraded, while in snow experiments, compounds with nitrogen, sulfur and phosphorus compounds were also degraded (Fig. 3a–b and f ,Tables 1, S4 and S5). These degraded nitrogen- and phosphorus-containing compounds were mainly oxygen-poor unsaturated aliphatics and highly unsaturated, while sulfur compounds included oxygen-rich unsaturated aliphatics and aromatics (Fig. 3f). Furthermore, ice samples had ~ 4 times more aromatics resistant to bio- and photo-degradation, while snow had ~ 3 times more highly bioavailable, unsaturated aliphatics (Figs. 1, 3 and S10a, Table 1). Degradation of hydrogen-rich aliphatics by heterotrophic processes (Fig. 3a and e–f), aligns with their bioavailability^30,43^ and potential utilization as a carbon source, being incorporated by heterotrophic microorganisms or transformed into new compounds.

After 22/24 days in darkness, heterotrophic metabolism resulted in a 74% (ice) and 24% (snow) increase in DOM-T0 formulae (Fig. 3c–d and g–h). In the ice, this increase was mainly in aromatics without heteroatoms related to oxy-aromatic phytochemicals, while in the snow this included nitrogen- and sulfur-containing compounds, partly related to oxy-aromatic phytochemicals (Fig. 3c–d and g–h, Tables 1, S4 and S5). Aromatic phytochemicals, have been related to cell signaling and stress resilience in microalgae^36^. Thus, their production in the blooms is expected and probably triggered by the extreme glacier conditions^4^. Accumulation of aromatic phytochemicals in the ice experiments indicates that they are not available for heterotrophic degradation (Fig. 3c), unlike the rapidly degraded sulfur-containing phytochemicals in the snow (Fig. 3f). Heterotrophic alteration removed labile DOM in both algal experiments. This degradation is expected to increase the abundance of refractory carboxyl-rich alicyclic compounds (CRAM)^45^. However, CRAM increased mainly in the snow, while in the ice some were heterotrophically degraded (Fig. 3a), suggesting microbial degradation of more refractory DOM in the ice habitat. Snow-algae blooms are earlier in the season and glacier ice-algae blooms peak later^4^. Our findings indicate that snow-algae provide bioavailable nitrogen-, sulfur- and phosphorus-bearing compounds that are quickly cycled by heterotrophs after snow melts. Although our experimental setup does not allow us to determine the origin of these compounds, the high abundance in our snow-algae photoproduced DOM (Fig. S8d) indicates that autotrophic bacteria or algae produce some of these bioavailable compounds (Fig. 3f). The 16S rRNA sequencing data (Fig. 3) confirmed a higher relative abundance of bacteria found in freshwater systems under the influence of ice melting and involved in iron cycling^46^ (ca. 23% of *Ferruginibacter*) and the ITS2 rRNA data (Fig. S11) revealed the abundance of cold adapted fungi (*Cryolevonia* and *Phenoliferia*) in the snow-algae habitat, suggesting a better adaptation to degrade more bioavailable snow-algae DOM. The ice-algae habitat was dominated by chemoautotrophs and ice-adapted genera (ca. 40% *Hymenobacter* and 22% *Parafrigobacterium*), which were present in snow at lower proportions (with ca. 26 and 12%, respectively). The high relative abundance of these microorganisms on the ice habitat suggest that they may be more versatile to degrade less bioavailable ice-algae DOM. Fungi such as Microbotryomycetes present higher relative abundance in the snow (Fig. S11). Their high relative abundance in our and other supraglacial habitats^47^ indicates that they are important OM decomposers^48^ and key players in algae-DOM alteration. Bacteria can also channel OM from primary production and in our samples sulfur oxidizers like *Acidophilum*^49^ may be responsible for the particular low abundance of sulfur compounds in the ice-algae habitat (Fig. 3). Moreover, arysulfatases make sulfate available to *Chlamydomonas* during periods of sulfate deficiency^27^. These enzymes may also occur in snow-algae potentially explaining sulfur degradation in the snow experiments (Fig. 3f), yet whether these change with environmental factors and bloom dynamics remains unknown. Cyanobacteria, which are dominant in cryoconite hole habitats, can also contribute to the overall surface ice OM pool when they become dispersed on the ice surfaces. However, in our initial glacier ice experimental sample cyanobacteria were present at below 0.7% in relative abundance at the genus level (included in “others” in Fig. 3), while in the snow sample they were fully absent. The most abundant cyanobacteria in our initial ice sample were the genus *Phormidesmis* (0.58%) and *Pseudanabaena (0.06%)*.

**Fig. 4:**
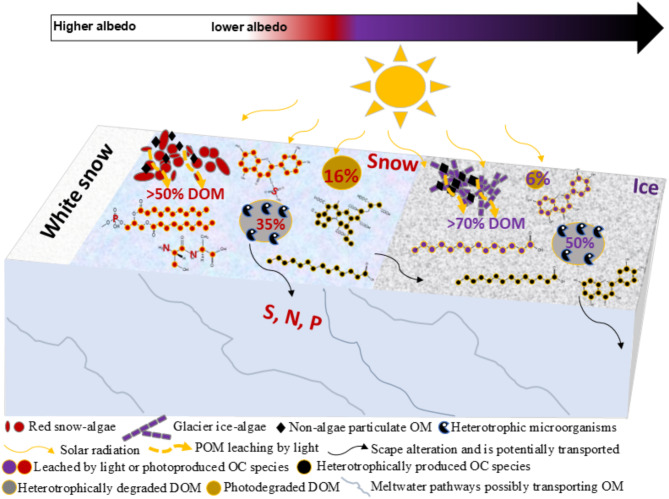
Conceptual diagram of the influence of light and heterotrophic processes on the OM composition of glacier ice- and snow-algae dominated habitats. As melting progresses, solar radiation refills > 50% of the DOM pool composition by stimulating autotrophic photoproduction and likely abiotic release of OM from POM, characterized in the red snow-algae habitat by higher diversity of heteroatoms compared to glacier ice-algae habitats. The red snow-algae habitat provides N-, S-, P-bearing compounds of unsaturated aliphatics, highly unsaturated and aromatics that are largely degraded by heterotrophic microorganisms that keep these compounds in low abundance after snow melts and bare-ice becomes predominant. Heterotrophic degradation removes 35 and 50% of the DOM found in red snow-algae and glacier ice-algae blooms, while solar radiation only 16 and 6%, respectively. The OM composition was strongly linked to the algae-dominating (glacier ice-algae or snow-algae) and influenced its degradation, resulting in an accumulation of resistant light-absorbing aromatics in glacier ice algae habitats (related to oxy-aromatic phytochemicals, black carbon or both) that can lead to more GrIS darkening (Table 1, Fig. S12).

Refractory OM, represented by formulae always present and not degraded with time, exhibited higher aromaticity in ice-algae compared to snow-algae habitats (Fig. S12). In ice, refractory OM also included unsaturated aliphatic and highly unsaturated primarily without heteroatoms, while in snow, they contained nitrogen and phosphorus (Fig. S12, Tables 1, S4 and S5). These distinctive snow- and ice-algae habitat signals can be either ice-locked during winter or released into streams in summer (Fig. 4). Aromatics associated to oxy-aromatic phytochemicals (43%) or BC (12%), were unique to ice habitats (Table 1 and Fig. S12), indicating that they play a bigger role in albedo reduction than in snow-algae habitats. This was, not only due to the role of glacial ice-algae phenolics and BC as light absorbers^18,50^ but due to their resistance to degradation and thus preferential accumulation. However, a detailed assessment of the role of pigmented glacier ice-algae blooms and BC on GrIS albedo reduction and to disentangle if these molecular signals are from atmospheric deposited BC, glacier ice-algae aromatic phytochemicals or both is still to be studied.

## Summary

Our findings highlight the compositional differences between OM in glacier ice-algae vs. red snow-algae dominated habitats and provides first insights into the GrIS microbial carbon dynamics in these biomes while, highlighting molecular changes due to light and heterotrophic processes. DOM alteration did not lead to the DOM pools becoming more similar as in marine settings^51^. Four-week-long incubations during the summer melt were insufficient to eliminate the very distinct molecular signals in the two different habitats. Glacier ice-algae DOM retained the higher abundance of highly bio-resistant aromatics associated with dissolved BC^15,19^, oxy-aromatic phytochemicals or both. In contrast, snow-algae DOM was dominated by unsaturated aliphatics richer in nitrogen-, sulfur-, phosphorus-bearing compounds supporting more pronounced heterotrophic alteration compared to ice-DOM. Despite the more refractory nature of glacier ice-algae DOM, POM was enriched in nitrogen-containing unsaturated aliphatics that were however, not transferred to the DOM pool during light exposure and thus were not bioavailable. Ice-DOM was initially less diverse than snow-DOM, but light exposure increased its complexity by triggering autotrophic photoproduction and abiotic POM leaching. Warmer climates will accelerate melting, increasing water availability further extending the areas for algal colonization and POM leaching. This in turn, may enhance OM release from such POM, providing labile DOM for further microbial alteration. This rise in labile DOM could support microbial and nutrient recycling^4^ stimulating new blooms, further altering the glacier carbon cycle. Autotrophic photoproduction in the snow-algae increased unique DOM-based sulfur- and phosphorus-bearing compounds that were partly heterotrophically degraded. Heterotrophic processes removed unsaturated aliphatics from the DOM pools in both habitats, increased aromatics in the ice and CRAM in the snow experiments. Aromatics and some unsaturated aliphatics may survive summer degradation and become frozen-in during diurnal freezing periods or retained by exopolymeric substances^41^, or they may be exported downstream with the meltwater^8,14^. Overall, our results and discussion indicate, on one hand that light-absorbing aromatics in glacial ice-algae habitats are not photochemically or heterotrophically degraded and thus they preferentially accumulate on the ice, likely decreasing the albedo. On the other hand, photochemical and heterotrophic degradation, which may help to increase the albedo, seem to have higher impact on the snow-algae habitat, were light-absorbing aromatics are removed (see Figs. S10 and S12). Thus, based on our experiments, the balance between these processes, suggest that glacier ice-algae habitats have a higher impact on altering glacier color and melting. Warmer conditions will extend bare-ice^16^, leading to more glacier ice-algae blooms with their higher abundance of light-absorbing and resistant aromatics in both DOM and POM pools that can further magnify GrIS darkening.

**Table 1:** Characteristics of the molecular formulae associated with solar radiation and heterotrophic processes.

	Transferred from POM to DOM due to solar radiation				Production				Degradation				Refractory	
					Solar radiation		Heterotrophic		Solar radiation		Heterotrophic			
	Glacier ice-algae habitat		Red snow-algae habitat		Glacier ice-algae habitat	Red snow-algae habitat	Glacier ice-algae habitat	Red snow-algae habitat	Glacier ice-algae habitat	Red snow-algae habitat	Glacier ice-algae habitat	Red snow-algae habitat	Glacier ice-algae habitat	Red snow-algae habitat
	Water soluble	Water insoluble	Water soluble	Water insoluble										
Number of molecularformulae	538	396	1112	1101	618	934	200	441	15	287	136	644	393	594
Molecular mass (Da)	432.3 ± 137.4	410.8 ± 129.7	512.0 ± 140.9	542.1 ± 130.8	473.6 ± 113.5	577.2 ± 138.1	390.1 ± 132.7	440.8 ± 142.1	421.9 ± 143.3	518.1 ± 100.4	352.4 ± 145.0	490.7 ± 146.1	354.6 ± 107.1	536.0 ± 128.4
CHO	93%	96%	61%	89%	92%	82%	93%	63%	80%	38%	92%	41%	92%	88%
CHON	6%	4%	22%	6%	6%	13%	5%	28%	13%	13%	6%	27%	8%	10%
CHOS	1%	0%	2%	1%	2%	3%	2%	8%	0%	33%	2%	20%	0%	0%
CHOP	1%	0%	13%	4%	0%	3%	1%	0%	7%	1%	0%	7%	0%	2%
O/C ratio	0.59 ± 0.34	0.58 ± 0.35	0.41 ± 0.17	0.36 ± 0.17	0.58 ± 0.34	0.36 ± 0.23	0.56 ± 0.46	0.42 ± 0.33	0.76 ± 0.34	0.28 ± 0.40	0.58 ± 0.34	0.36 ± 0.37	0.45 ± 0.44	0.39 ± 0.16
H/C ratio	1.09 ± 0.15	1.07 ± 0.15	1.50 ± 0.11	1.52 ± 0.10	1.07 ± 0.15	1.42 ± 0.13	0.96 ± 0.17	1.36 ± 0.22	0.97 ± 0.16	1.41 ± 0.23	1.07 ± 0.17	1.47 ± 0.21	1.30 ± 0.15	1.49 ± 0.09
Aromaticity index (Aimod)	0.31 ± 0.23	0.34 ± 0.23	0.00 ± 0.11	0. 00 ± 0.09	0.32 ± 0.23	0.04 ± 0.15	0.43 ± 0.29	0.10 ± 0.21	0.33 ± 0.28	0.15 ± 0.22	0.32 ± 0.22	0.17 ± 0.21	0.26 ± 0.26	0.13 ± 0.10
Nominal oxidation state of carbon (NOSC)	0.1 ± 0.45	0.1 ± 3.47	-0.6 ± 0.35	-0.8 ± 0.31	0.1 ± 0.44	-0.7 ± 0.30	0.2 ± 0.57	-0.4 ± 0.43	0.6 ± 0.25	-0.7 ± 0.47	0.12 ± 0.51	-0.55 ± 0.51	-0.36 ± 0.60	-0.69 ± 0.26
DBE-O	-1.2 ± 3.76	-0.6 ± 0.48	-2.1 ± 3.73	-2.0 ± 2.82	-1.3 ± 4.17	-0.8 ± 5.23	0.7 ± 4.54	0.6 ± 7.13	-2.0 ± 4.93	3.4 ± 9.87	-1.3 ± 4.11	1.1 ± 9.23	-0.8 ± 3.57	-2.3 ± 2.94
Aromatics	22%	25%	0%	0%	23%	4%	49%	10%	33%	15%	23%	14%	22%	0%
Condensed aromatics	9%	10%	0%	0%	8%	1%	32%	3%	13%	9%	8%	4%	12%	0%
Aliphatics	13%	12%	53%	54%	11%	39%	18%	33%	13%	39%	11%	55%	39%	46%
Aliphatics with nitrogen	2%	1%	14%	3%	1%	9%	1%	14%	7%	5%	1%	19%	1%	4%
Phytochemicals	73%	76%	10%	11%	72%	22%	70%	37%	80%	25%	72%	21%	43%	11%
CRAM	40%	41%	33%	31%	44%	36%	22%	32%	7%	24%	44%	28%	30%	39%

Aromaticity index (AImod) is = 1 + C-0.5O-S-0.5(H + N + P))/(C-0.5O-S-N-P) (where C, H, N, P and S are carbon, hydrogen nitrogen, phosphorus and sulphur, respectively). Nominal oxidation state of carbon (NOSC) = 4-(4 C + H-3 N-2O-2 S)/C.

### Online methods

#### Field sampling and experimental setup

In summer 2021, surface red snow- or purple glacier ice-algae dominated samples (1–2 millimeters for snow and max five centimeters for ice) were collected using a metal trowel and metal ice axe immersed in each respective habitat prior to sampling (Fig. S1). The sampling was done in July (Fig. S2) at the southern tip of the GrIS, upwind of the DEEP PURPLE ice camp (deeppurple-ercsyg.eu) located at 61°6’ N, 46°51’ W at 628 m altitude and about 600 m NW of the QAS-M Promice weather station (promice.org). In our study site, active melting occurred, as documented by the relatively high air temperatures recorded by the QAS-M meteorological station instruments (Fig. S2a). Our findings also match data from another recent study that evaluated OM cycling in the weathering crust at a site near to our sample location^12^. For our study, we collected samples in areas that were cryoconite free, and this is also confirmed by the low levels of cyanobacteria in our 16S rRNA amplicon data (see results and discussion, and caption of Fig. 3). From each habitat, ~ 24 L of snow or ice were collected into sterile Whirl-Pak bags which were slowly thawed (~ 1 day) inside a dedicated solvent-free tent. Once thawed, the material was homogenized and, for each experiment, 200 mL were transferred into vented Falcon bottles (four per time point), without the addition of nutrients to simulate oligotrophic conditions. These were either left in full light or fully covered with aluminum foil (represented by the four light and three dark bottles in Fig. S1a). The light radiation passing through the incubation bottles was between 350 and 800 nm, thus including the upper wavelengths of the UV-A but excluding UV-B. Accordingly, the photochemical effects in our experiments can be considered conservative and have the potential to be larger than we demonstrate in this study. All bottles were left on the ice for up to 24 days in an experimental plot (Fig. S1b). In addition, from each of the two sampling sites, a subset each of snow and ice, representing the initial time point (T0, ~ 1 L), was collected in muffled glass jars, in order to be processed immediately after thawing (~ 15 h) and avoid potential contamination. To evaluate potential contamination during incubation and sample preparation, Milli-Q water from the home laboratory was also incubated in six light and dark Falcon bottle pairs. It should be noted that our 24-day experiments were carried out with melted and homogeneized snow and ice samples. Thus, the snow and ice matrix effects on the transmitted and diffuse solar radiance could not be capture.

#### Microbial community composition and organic matter analysis

From the initial snow and ice material additional 250 mL were melted and filtered, and flash-frozen (− 80°C) for genomic analyses. DNA was extracted using the PowerSoil Pro kit (QIAgen) according to the manufacturer’s protocol. We used the 16S rRNA procaryotic primer pair gene Bakt_341F (CCTACGGGNGGCWGCAG) and Bakt_805R (GACTACHVGGGTATCTAATCC)^52^, the 18S rRNA eukaryotic primer pair 528F (5’-GCGGTAATTCCAGCTCCAA-3‘) and 706R (5’-AATCCRAGAATTTCACCTCT-3‘)^53^, and the internal transcribed spacer 2 (ITS2 snow) gene primers 5.8SbF (5′-GATGAAGAACGCAGCG-3′)^54^ and ITS4R (5′-TCCTCCGCTTATTGATATGC-3′)^55^ and the ITS2 ice primers 5.8SbF (5’-CGATGAAGAACGCAGCG-3’) and LSULP (5’-AATTCGGCGGGTGGTCTTG-3’)^22^ for PCR amplification. The resulting libraries were sequenced on an Illumina MiSeq using the V2 kit (Illumina Inc. SanDiego, California, US) resulting in 2 × 250 bp reads. Full details of sequencing and data processing can be found in previous publications^24,56^.

At specific time points (between 3 and 24 days, Fig. S1a), pairs of light and dark Falcon bottles were removed from the experimental plot and the contents filtered through pre-combusted 0.7 μm GF/F filter (Whatman) using an acid washed glass filtration unit. The particulates retained in the GF/F filters were packed in ashed aluminum foil, frozen in a portable − 20 freezer, and returned to the home laboratory frozen. The resulting solutions were retained in a 1 L glass bottle and acidified to pH 2 with HCl 37% (Aristar, VWR for trace metals). An aliquot of this acidified solution was transferred into pre-combusted glass amber vials for DOC analysis and the remainder was solid phase extracted (SPE) on 1 g PPL cartridges as previously described^57^. The DOC concentration in the amber vials was analyzed in the home laboratory using catalytic oxidation at high temperature on a TOC-V Shimadzu instrument^58^. To assess the POM components, the particulates retained in the GF/F filters were extracted with both hot water (POM-HW) and sodium hydroxide (POM-NaOH), representing water-soluble and water-insoluble OC, respectively, following the methods described in Antony et al.^59^ in review. The DOC concentrations in these extracts were also analyzed^58^ and subsequently SPE extracted as described above. All SPE extracts were eluted from the PPL cartridges with methanol and diluted in 1:1 methanol: ultrapure water to a final DOC concentration of 5 mg C L^− 1^ for untargeted molecular characterization on a Solarix 15T FTICR-MS (Bruker Daltonic) equipped with an electrospray ionization source (ESI, Bruker Apollo II) in negative ion mode. The mass spectrometric approach employed in this study allowed us to detect a wide range polar and acidic compounds within the DOM and POM pools but did not allow us to distinguish specific compounds. This is particularly true for pigments, which are primarily carotenoids in snow samples^6^ and phenolic purpurogallin type pigments in the ice samples^29^. In our data, water-soluble derivatives of pigments, which may have OH, COOH and carbohydrates groups, are likely present in our analytical window and are contained in different molecular categories such as aromatics (e.g., phenolic aromatics) and highly unsaturated (e.g., purpurogallin carboxylic acid^29^), some of which can fall in the category of phytochemicals if they have the previously defined elemental ratios^34^.

The obtained extracts were analyzed in duplicates. The extracts were infused at 120 µL h^− 1^ and the ions were accumulated in the hexapole for 0.2 s prior to ICR cell, with individual spectra obtained after 200 individual scans. External and internal calibrations were performed based on arginine cluster and known molecular mass peaks detected over the whole mass range (100–1000 m/z). Molecular formula calculation was performed using the software ICBM-OCEAN^60^ that allows to remove the noise based on the method detection limit, align the different mass spectra and calculate molecular formulae using an homologous series network approach (CH_2_, CO_2_, H_2_, H_2_O, and O). Molecular formulae above the method detection limit (MDL) 2 were assigned using a sample junction in fast join mode and a recalibration tolerance of 0.5 ppm. Minimum signal to MDL ratio as backbone for recalibration was 5 using mean recalibration mode. Molecular formulae assigned excluded isotope ratio mismatches above signal to MDL ratios of isotope formulae > 5. Isotope tolerance was set to 1000‰. For the assignments the following combinations of elements were allowed: C_0 − 100_, O_0 − 50_, H_0 − 200_, N_0 − 4_, S_0 − 2_ and P_0 − 1_. Mass peaks (10620) used for calculations of relative abundances of our molecular formulae were obtained after all peaks present in the Milli-Q water blanks were removed from the dataset. Because samples were analyzed in duplicates, a compound was considered to be present if it appeared in both duplicate measurements and their mean normalized intensity was used for statistical analysis. Following this approach, 8827 molecular formulae were obtained for the whole dataset, not considering isotopic peaks^61^. In order to visualize the formulae that were characteristic of each habitat, van Krevelen diagrams (VKD) with H/C and O/C ratio were produced using the NMDS molecular loadings (i.e., variation of grey crosses along the X axis from Fig. 1). Similarly, VKD are presented to highlight the compounds that change over the experiment due to solar radiation (Figs. 2, S7-S10) and heterotrophic processes (Fig. 3). Autotrophic OM production was considered as the progressive increase in relative abundance of molecular formulae exclusively in light conditions, while heterotrophic OM production or degradation was inferred to be the progressive increase or decrease in relative abundance of formulae in the experiments under dark conditions, although not exclusively.

Structural information for all molecular formulae was obtained based on several ratios and indices previously described^60^ and in the supplementary material. Mean values for the double bond equivalent (DBE), DBE-O (minus oxygen) and the aromaticity index (AImod), as well as H/C and O/C elemental ratios and the nominal oxidation state of carbon (NOSC) were calculated considering the intensity of the formulae peak in each sample (DBE-O_wa_, AImod_wa_, NOSC_wa_, H/C_wa_ and O/C_wa_, Table S6). Average values were calculated for the formulae associated with the processes described in this study (Table 1). To obtain an overview of the molecular formulae distribution and their potential variations in the experiments, formulae were furthermore associated with different molecular categories based on the elemental ratios, heteroatom compositions (nitrogen, sulfur and phosphorous) and AImod (Table S7) following previous FTICR-MS studies^34,60^. These categories only indicate that the formula is the same of a known molecule although its chemical structure may differ.

## Electronic supplementary material

Below is the link to the electronic supplementary material.

Supplementary Material 1.

Supplementary Material 2.

## Data Availability

All FTICR-MS and DOC data have been deposited in the GFZ data repository with the links: 10.5880/GFZ.3.5.2024.002 and 10.5880/GFZ.YOGU.2025.001 and is further available in the supplementary tables. Sequence data is available on NCBI SRA under the accession number PRJNA1209915 for the 18S rRNA gene sequencing and PRJNA1209368 for the 16S rRNA gene sequencing. For the amplicon data related to the initial red snow- and glacier ice-algae samples used in the experiments see the links: https://www.ncbi.nlm.nih.gov/sra/?term=SAMN46219422 and https://www.ncbi.nlm.nih.gov/sra/?term=SAMN46219445, respectively.

## References

[1] Hodson, A. et al. Glacial ecosystems. *Ecol. Monogr.* **78**, 41–67 (2008).

[2] Nghiem, S. V. et al. The extreme melt across the Greenland ice sheet in 2012. *Geophys. Res. Lett.* **39** (2012).

[3] Van den Hurk, B. et al. Fact Sheet Relevant for Sector – Water Resources Management. In *Climate Change 2021: The Physical Science Basis,* (eds Masson-Delmotte, V., Zhai, P., Pirani, A., Connors, S. L., Péan, C., Berger, S., Caud, N., Chen, Y., Goldfarb, L., Gomis, M. I., Huang, M., Leitzell, K., Lonnoy, E., Matthews, J. B. R., Maycock, T. K., Waterfield, T., Yelekçi, O., Yu, R., Zhou, B.) Contribution of Working Group I to the Sixth Assessment Report of the Intergovernmental Panel on Climate Change (IPCC).

[4] Anesio, A. M., Lutz, S., Chrismas, N. A. M. & Benning, L. G. The microbiome of glaciers and ice sheets. *Npj Biofilms Microbiomes*. **3**, 10 (2017). 28649411 10.1038/s41522-017-0019-0 PMC5460203

[5] Chevrollier, L. A. et al. Light absorption and albedo reduction by pigmented microalgae on snow and ice. *J. Glaciol.* **69**, 333–341 (2023).

[6] Lutz, S. et al. The biogeography of red snow microbiomes and their role in melting Arctic glaciers. *Nat. Commun.* **7**, 11968 (2016). 27329445 10.1038/ncomms11968 PMC4917964

[7] Yallop, M. L. et al. Photophysiology and albedo-changing potential of the ice algal community on the surface of the Greenland ice sheet. *ISME J.* **6**, 2302–2313 (2012). 23018772 10.1038/ismej.2012.107 PMC3504962

[8] Musilova, M. et al. Microbially driven export of labile organic carbon from the Greenland ice sheet. *Nat. Geosci.* **10**, 360–365 (2017).

[9] Andrews, M. G., Jacobson, A. D., Osburn, M. R. & Flynn, T. M. Dissolved carbon dynamics in meltwaters from the Russell glacier, Greenland ice sheet. *J. Geophys. Res. Biogeosci.* **123**, 2922–2940 (2018).

[10] Anesio, A. M. et al. Carbon fluxes through bacterial communities on glacier surfaces. *Ann. Glaciol.* **51**, 32–40 (2010).

[11] Bhatia, M. P., Das, S. B., Longnecker, K., Charette, M. A. & Kujawinski, E. B. Molecular characterization of dissolved organic matter associated with the Greenland ice sheet. *Geochim. Cosmochim. Acta* **74**, 3768–3784 (2010).

[12] Doting, E. L. et al. Molecular level characterization of supraglacial dissolved organic matter sources and exported pools on the Southern Greenland ice sheet. *Biogeosciences* **22**, 41–53 (2025).

[13] Lawson, E. C. et al. Greenland ice sheet exports labile organic carbon to the Arctic oceans. *Biogeosciences* **11**, 4015–4028 (2014).

[14] Hood, E. et al. Glaciers as a source of ancient and labile organic matter to the marine environment. *Nature* **462**, 1044–1047 (2009). 20033045 10.1038/nature08580

[15] Singer, G. A. et al. Biogeochemically diverse organic matter in alpine glaciers and its downstream fate. *Nat. Geosci.* **5**, 710–714 (2012).

[16] Cook, J. M. et al. Glacier algae accelerate melt rates on the south-western Greenland ice sheet. *Cryosphere* **14**, 309–330 (2020).

[17] Lutz, S., McCutcheon, J., McQuaid, J. B. & Benning, L. G. The diversity of ice algal communities on the Greenland ice sheet as revealed by oligotyping. *Microb. Genomics* **4** (2018). 10.1099/mgen.0.000159 PMC5885011 29547098

[18] Williamson, C. J. et al. Algal photophysiology drives darkening and melt of the Greenland Ice Sheet. *Proc. Natl. Acad. Sci.* **117**, 5694–5705 (2020). 10.1073/pnas.1918412117 PMC7084142 32094168

[19] Stubbins, A. et al. Anthropogenic aerosols as a source of ancient dissolved organic matter in glaciers. *Nat. Geosci.* **5**, 198–201 (2012).

[20] Kellerman, A. M. et al. Molecular signatures of glacial dissolved organic matter from Svalbard and Greenland. *Glob. Biogeochem. Cycles* **35**, e2020GB006709 (2021).

[21] Álvarez-Salgado, X. A., Nieto-Cid, M. & Rossel, P. E. Dissolved organic matter. in Marine Analytical Chemistry (eds Blasco, J. & Tovar-Sánchez, A.) 39–102 (Springer International Publishing, Cham, 2023). 10.1007/978-3-031-14486-8_2.

[22] Remias, D., Procházková, L., Nedbalová, L., Benning, L. G. & Lutz, S. Novel insights in cryptic diversity of snow and glacier ice algae communities combining 18S rRNA gene and ITS2 amplicon sequencing. *FEMS Microbiol. Ecol.* **99**, fiad134 (2023). 37880981 10.1093/femsec/fiad134 PMC10659120

[23] Hotaling, S. et al. Biological albedo reduction on ice sheets, glaciers, and snowfields. *Earth-Sci. Rev.* **220**, 103728 (2021).

[24] Peter, E. K. et al. Endometabolic profiling of pigmented glacier ice algae: The impact of sample processing. *Metabolomics* **20**, 98 (2024). 39123092 10.1007/s11306-024-02147-6 PMC11315761

[25] McCutcheon, J. et al. Mineral phosphorus drives glacier algal blooms on the Greenland ice sheet. *Nat. Commun.* **12**, 570 (2021). 33495440 10.1038/s41467-020-20627-w PMC7835244

[26] Slocombe, S. P. et al. Overexpression of PSR1 in Chlamydomonas reinhardtii induces luxury phosphorus uptake. *Front. Plant. Sci.* **14**, (2023). 10.3389/fpls.2023.1208168 PMC10413257 37575910

[27] Giordano, M., Norici, A., Ratti, S. & Raven, J. A. Role of sulfur for algae: Acquisition, metabolism, ecology and evolution. in Sulfur Metabolism in Phototrophic Organisms (eds Hell, R., Dahl, C., Knaff, D. & Leustek, T.) 397–415 (Springer Netherlands, Dordrecht, 2008). 10.1007/978-1-4020-6863-8_20.

[28] Rezayian, M., Niknam, V. & Ebrahimzadeh, H. Oxidative damage and antioxidative system in algae. *Toxicol. Rep.* **6**, 1309–1313 (2019). 31993331 10.1016/j.toxrep.2019.10.001 PMC6978204

[29] Remias, D. et al. Characterization of an UV- and VIS-absorbing, purpurogallin-derived secondary pigment new to algae and highly abundant in*Mesotaenium berggrenii* (Zygnematophyceae, Chlorophyta), an extremophyte living on glaciers. *FEMS Microbiol. Ecol.* **79**, 638–648 (2012). 22092588 10.1111/j.1574-6941.2011.01245.x

[30] D’Andrilli, J., Cooper, W. T., Foreman, C. M. & Marshall, A. G. An ultrahigh-resolution mass spectrometry index to estimate natural organic matter lability. *Rapid Commun. Mass. Spectrom.* **29**, 2385–2401 (2015). 26563709 10.1002/rcm.7400 PMC4654268

[31] Feord, H. et al. G. A novel approach for cryobiome functional analysis with metaproteomics. (Vienna, Austria, 2023). 10.5194/egusphere-egu23-14504.

[32] Lutz, S., Anesio, A. M., Field, K. & Benning, L. G. Integrated ‘omics’, targeted metabolite and Single-cell analyses of Arctic snow algae functionality and adaptability. *Front. Microbiol.* **6** (2015). 10.3389/fmicb.2015.01323 PMC4659291 26635781

[33] Hadley, O. L. & Kirchstetter, T. W. Black-carbon reduction of snow albedo. *Nat. Clim. Change*. **2**, 437–440 (2012).

[34] Rivas-Ubach, A. et al. Moving beyond the Van Krevelen diagram: A new stoichiometric approach for compound classification in organisms. *Anal. Chem.* **90**, 6152–6160 (2018). 29671593 10.1021/acs.analchem.8b00529

[35] Freile-Pelegrín, Y. & Robledo, D. Bioactive phenolic compounds from algae. In *Bioactive Compounds from Marine Foods* 113–129 (Wiley, Ltd, 2013). 10.1002/9781118412893.ch6.

[36] Goiris, K. et al. Detection of flavonoids in microalgae from different evolutionary lineages. *J. Phycol.* **50**, 483–492 (2014). 26988321 10.1111/jpy.12180

[37] Halbach, L. et al. Pigment signatures of algal communities and their implications for glacier surface darkening. *Sci. Rep.* **12**, 17643 (2022). 36271236 10.1038/s41598-022-22271-4 PMC9587043

[38] Antony, R. et al. Photo-biochemical transformation of dissolved organic matter on the surface of the coastal East Antarctic ice sheet. *Biogeochemistry* **141**, 229–247 (2018).

[39] Feng, L. et al. Molecular insights into glacial cryoconite dissolved organic matter evolution under dark conditions during the ablation season on the Tibetan plateau. *ACS Earth Space Chem.* **5**, 870–879 (2021).

[40] Hood, E., Battin, T. J., Fellman, J., O’Neel, S. & Spencer, R. G. M. Storage and release of organic carbon from glaciers and ice sheets. *Nat. Geosci.* **8**, 91–96 (2015).

[41] Lutz, S., Anesio, A. M., Villar, J., Benning, L. G. & S. E. & Variations of algal communities cause darkening of a Greenland glacier. *FEMS Microbiol. Ecol.* **89**, 402–414 (2014). 24920320 10.1111/1574-6941.12351

[42] Cory, R. M. & Kling, G. W. Interactions between sunlight and microorganisms influence dissolved organic matter degradation along the aquatic continuum. *Limnol. Oceanogr. Lett.* **3**, 102–116 (2018).

[43] Antony, R. et al. Molecular insights on dissolved organic matter transformation by supraglacial microbial communities. *Environ. Sci. Technol.* **51**, 4328–4337 (2017). 28328192 10.1021/acs.est.6b05780

[44] Mopper, K., Kieber, D. J. & Stubbins, A. *Marine Photochemistry of Organic Matter: Processes and Impacts. Biogeochemistry of Marine Dissolved Organic Matter* (Elsevier Inc., 2015). 10.1016/B978-0-12-405940-5.00008-X.

[45] Hertkorn, N. et al. Characterization of a major refractory component of marine dissolved organic matter. **70**, 2990–3010 (2006).

[46] Papale, M. et al. Benthic Microbial communities in a seasonally ice-covered Sub-Arctic river (Pasvik River, Norway) are shaped by site-specific environmental conditions. *Microorganisms* **10** (2022). 10.3390/microorganisms10051022 PMC9147904 35630464

[47] Perini, L., Andrejašič, K., Gostinčar, C., Gunde-Cimerman, N. & Zalar, P. Greenland and Svalbard glaciers host unknown basidiomycetes: The yeast *Camptobasidium arcticum* Sp. Nov. And the dimorphic *Psychromyces glacialis* gen. and Sp. nov. *Int. J. Syst. Evol. MicroBiol.* **71** (2021). 10.1099/ijsem.0.004655 PMC8346769 33502296

[48] Sanderman, J. & Amundson, R. Biogeochemistry of decomposition and detrital processing. *Biogeochemistry* **10** (2014).

[49] Dahl, C. Inorganic sulfur compounds as electron donors in purple sulfur bacteria. in Sulfur Metabolism in Phototrophic Organisms (eds Hell, R., Dahl, C., Knaff, D. & Leustek, T.) 289–317 (Springer Netherlands, Dordrecht, 2008) 10.1007/978-1-4020-6863-8_15.

[50] Stubbins, A. et al. Utilizing colored dissolved organic matter to derive dissolved black carbon export by Arctic rivers. *Front. Earth Sci.* **3**, (2015).

[51] Mentges, A., Feenders, C., Seibt, M., Blasius, B. & Dittmar, T. Functional molecular diversity of marine dissolved organic matter is reduced during degradation. *Front. Mar. Sci.* **4**, (2017).

[52] Herlemann, D. P. R. et al. Transitions in bacterial communities along the 2000 Km salinity gradient of the Baltic sea. *ISME J.* **5**, 1571–1579 (2011). 21472016 10.1038/ismej.2011.41 PMC3176514

[53] Cheung, M. K., Au, C. H., Chu, K. H., Kwan, H. S. & Wong, C. K. Composition and genetic diversity of Picoeukaryotes in subtropical coastal waters as revealed by 454 pyrosequencing. *ISME J.* **4**, 1053–1059 (2010). 20336159 10.1038/ismej.2010.26

[54] Mikhailyuk, T. I. et al. New streptophyte green algae from terrestrial habitats and an assessment of the genus interfilum (KLEBSORMIDIOPHYCEAE, STREPTOPHYTA). *J. Phycol.* **44**, 1586–1603 (2008). 27039871 10.1111/j.1529-8817.2008.00606.x

[55] White, T. et al. Amplification and Direct Sequencing of Fungal Ribosomal RNA Genes for Phylogenetics. In *PCR Protocols: a Guide to Methods and Applications* vol. 31 (1990).

[56] Mourot, R. Ecology of Supraglacial Microbial Communities. (Freie Universität Berlin).

[57] Dittmar, T., Koch, B., Hertkorn, N. & Kattner, G. A simple and efficient method for the solid-phase extraction of dissolved organic matter (SPE-DOM) from seawater. *Limnol. Oceanogr. Methods* 230–235 (2008).

[58] Rossel, P. E. et al. Dissolved organic carbon concentrations from the dissolved organic matter (DOM) and extracted particulate OM (POM) obtained from purple glacier ice- and red snow-algae dominated surface habitats collected close to the QAS-M Promice weather station on the Southern tip of the Greenland ice sheet. *GFZ Data Serv.*10.5880/GFZ.YOGU.2025.001 (2025).

[59] Antony, R. et al. Extraction strategies for profiling the molecular composition of particulate organic matter on glacier surfaces. *Environ. Sci. Tech.*10.1021/acs.est.4c10088 (2025). 10.1021/acs.est.4c10088 PMC11912203 40016117

[60] Merder, J. et al. ICBM-OCEAN: processing Ultrahigh-Resolution mass spectrometry data of complex molecular mixtures. *Anal. Chem.* **92**, 6832–6838 (2020). 32298576 10.1021/acs.analchem.9b05659

[61] Rossel, P. E. et al. Normalized peak intensities obtained from the molecular analysis of organic matter from purple glacier ice- and red snow-algae dominated surface habitats collected close to the QAS-M Promice weather station on the Southern tip of the Greenland ice sheet. *GFZ Data Serv.*10.5880/GFZ.3.5.2024.002 (2025).

