# Author Correction: Dynamics of organic matter in algal blooms on the Greenland ice sheet

**DOI:** 10.1038/s41598-025-96992-7

**Published:** 2025-06-04

**Authors:** Pamela E. Rossel, Runa Antony, Rey Mourot, Thorsten Dittmar, Alexandre M. Anesio, Martyn Tranter, Liane G. Benning

**Affiliations:** 1 Interface Geochemistry Section, GFZ Helmoltz Centre for Geosciences, Potsdam, Germany; 2 National Centre for Polar and Ocean Research, Ministry of Earth Sciences, Vasco da Gama, Goa India; 3 Aix Marseille Univ, Université de Toulon, CNRS, IRD, MIO, Marseille, France; 4 Institute for Chemistry and Biology of the Marine Environment (ICBM), Carl Von Ossietzky University Oldenburg, Oldenburg, Germany; 5 Helmholtz Institute for Functional Marine Biodiversity (HIFMB), Oldenburg, Germany; 6 Department of Environmental Science, Aarhus University, Frederiksborgvej 399, 4000 Roskilde, Denmark; 7 Department of Earth Sciences, Freie Universität Berlin, 12249 Berlin, Germany

Correction to: *Scientific Reports* 10.1038/s41598-025-92182-7, published online 10 March 2025

The Funding section in the original version of this Article was incomplete.

Open Access funding enabled and organized by Projekt DEAL.

now reads:

This study is funded by the European Research Council (ERC) Synergy Grant DEEP PURPLE under the European Union’s Horizon 2020 research and innovation programme (Grant agreement No 856416). Open Access funding enabled and organized by Projekt DEAL.

The original Article has been corrected.

