# Supplementary material for: Dynamics of organic matter in algal blooms on the Greenland ice sheet
Source: Sci Rep. 2025 Mar 10;15:8288. doi: 10.1038/s41598-025-92182-7 (PMC11893808; doi:10.1038/s41598-025-92182-7)
Supplement: Supplementary file 2 — Supplementary Material 2. [file 41598_2025_92182_MOESM2_ESM.docx]

Dynamics of organic matter in algal blooms on the

Greenland Ice Sheet

Pamela E. Rossel^1^, Runa Antony^1,2^, Rey Mourot^1,3^, Thorsten Dittmar^4,5^, Alexandre M. Anesio^6^, Martyn Tranter^6^ and Liane G. Benning^1,7^

**In review for Sci. Reports**

^1^ Interface Geochemistry Section, GFZ Helmholtz Centre for Geosciences, Potsdam, Germany.

^2^National Centre for Polar and Ocean Research, Ministry of Earth Sciences, Goa, India

^3^Aix Marseille Univ, Université de Toulon, CNRS, IRD, MIO, Marseille, France

^4^Institute for Chemistry and Biology of the Marine Environment (ICBM), Carl von Ossietzky University Oldenburg, Oldenburg, Germany

^5^Helmholtz Institute for Functional Marine Biodiversity (HIFMB), Oldenburg, Germany

^6^Department of Environmental Science, Aarhus University, Frederiksborgvej 399, 4000 Roskilde, Denmark

^7^Department of Earth Sciences, Freie Universität Berlin, 12249 Berlin, Germany

(*correspondence: [prossel@gfz.de](mailto:prossel@gfz..de)).

Supplementary Note 1:

Estimation of the OM associated to algae cell in high biomass ice

To obtain the carbon equivalent biomass, average glacier algal cell volume (3370 µm^3^)^1^, and carbon mass conversion constants (0.109 and 0.991)^2^ previously reported were used according to the follow equation:

$$\boldsymbol{y=a}\boldsymbol{x}^{\boldsymbol{b}}$$

Where Y is the C content (pg C/cell), x is the cell volume (µm^3^) and a and b are the constants (0.109 and 0.991, respectively)

$\boldsymbol{y=0.109\times}\boldsymbol{3370}^{\boldsymbol{0.991}}$ , Thus, carbon equivalent biomass is 341.44 pg C/cell

Using average total organic carbon (TOC) concentration in high biomass ice (20.5 mg C/L) and glacial algal cells abundance after removing bacteria and black carbon contribution to TOC (5.63x10^4^ cells/ml)^1^, we calculated TOC in algal cells and their contribution to total TOC reported in these high biomass habitats as follows:

$\boldsymbol{TOCcells=341}\boldsymbol{pgC\times5.63}\boldsymbol{x}\boldsymbol{10}^{\boldsymbol{4}}\boldsymbol{cells}/\boldsymbol{ml}$, Thus, TOC from algal cells is 19.2 mg C/L.

This is 93.6% of the TOC in high biomass ice and we know from our data that DOC (~1 mg C/L) represents a minor fraction (< 9%) of the TOC.


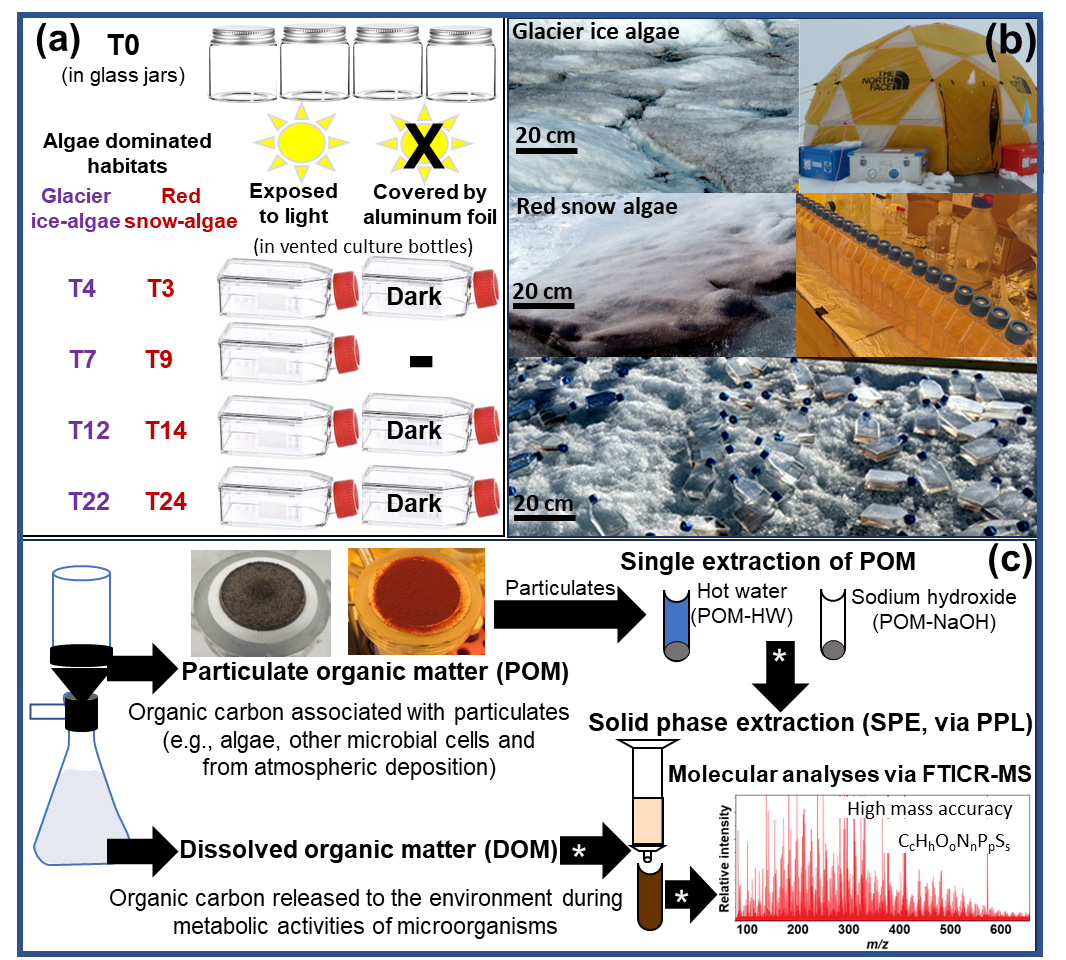


**Figure S1. Experimental setup of glacier ice- and red snow-algae samples and organic matter processing.** (**a**) Experimental setup indicating incubation time in days for the experiments performed under light and dark conditions with the whole material collected in each habitat (except for T0 processed after thawing), (**b**) habitats dominated by glacier ice- and red snow-algae sampled for the experiments, solvent-free tent used for experimental setup prior to incubation on the ice/snow surface and (**c**) sample processing after time point completion (performed in the field for the DOM and in the laboratory for POM). Particulates were extracted with hot water (POM-HW) and sodium hydroxide (POM-NaOH) to represent water-soluble and insoluble organic compounds, respectively (see methods). Analysis of DOC concentrations during sample processing is indicated by * in (**c**). Picture of incubated bottles courtesy of C. Trivedi.


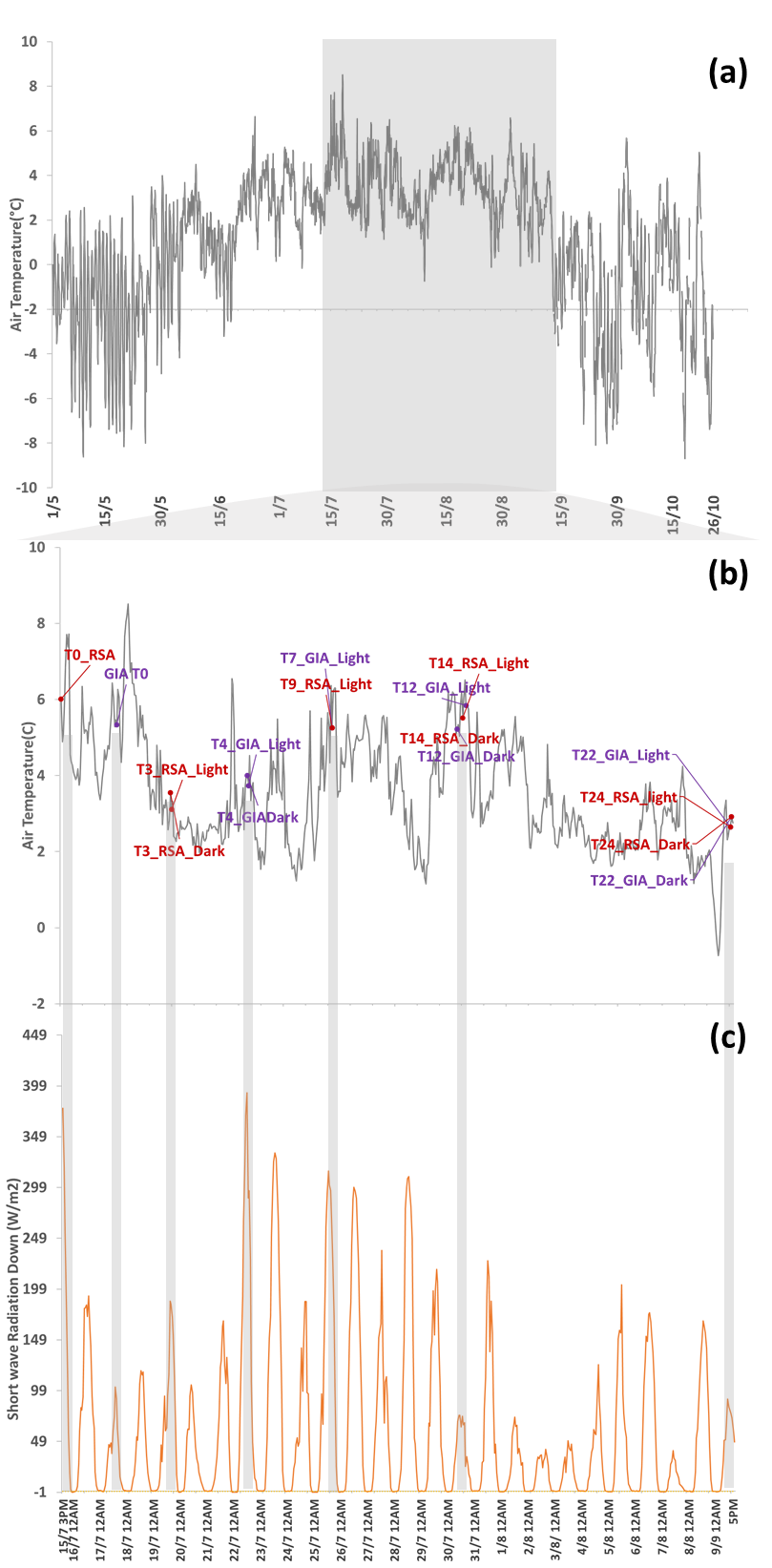


**Figure S2. Air temperature and shortwave radiation down monitored by the PROMICE automatic weather station QAS_M.** (**a)** Air temperatures during May 1st to October 25th 2021, highlighting in grey the 24 day period of the incubation experiments, and (**b)** and (**c)** air temperatures and short-wave radiation down with its 24h cycles, respectively, over the experimental period; marked with grey bars are the time point when pairs of dark and light glacier ice-algae (GIA, in purple) and red snow-algae (RSA, in dark red) sample bottles were removed from the experimental plot. Note: light variations did not affect dark experiments because the associated bottles were fully covered by aluminum foil.

**
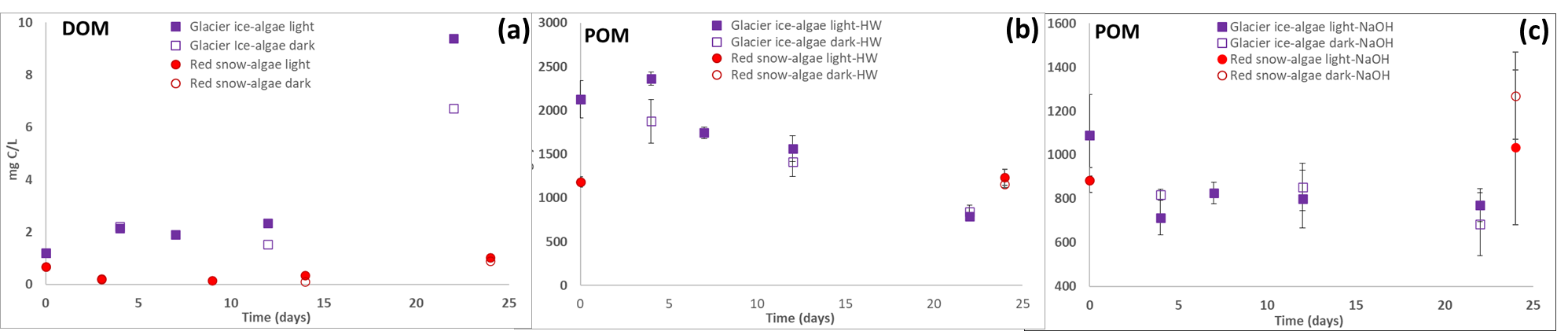
**

**Figure S3. Dissolved organic concentration of DOM and POM pools.** (**a**) DOC concentrations in the DOM pool and POM represented as (**b**) water-soluble OM extracted with hot water (HW) and (**c**) water-insoluble OM extracted with NaOH for glacier ice- and red snow-algae experiments performed under light and dark conditions. Error bars represent the standard variation of replicate measurements. Error bars for DOC measured in DOM pool are smaller than the symbols.


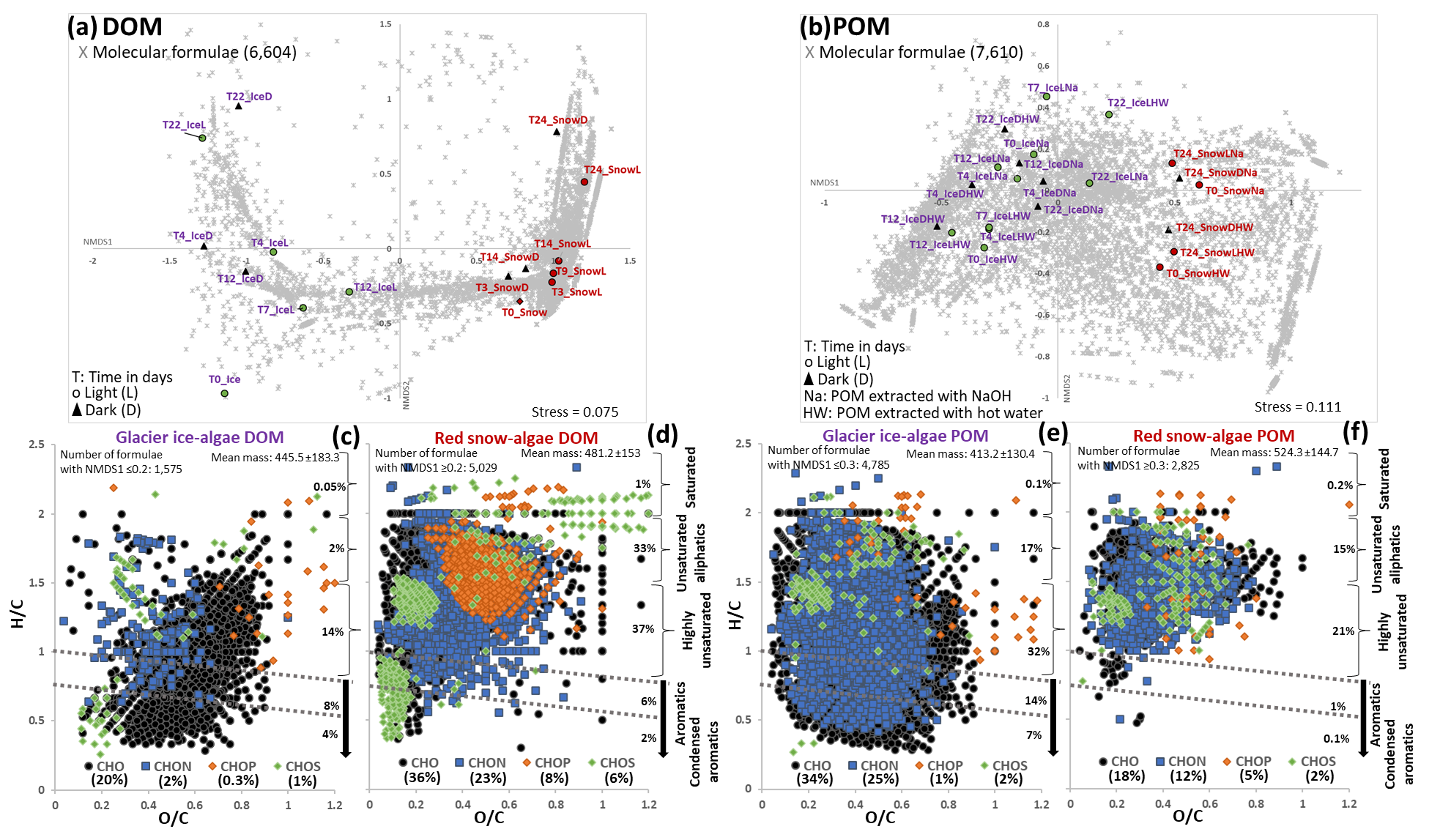


**Figure S4. Compositional differences in the DOM and POM pools on the surface of glacier ice-algae and red snow-algae dominated habitats based on Bray Curtis dissimilarity.** (**a**) Non-metric multidimensional scaling (NMDS) of DOM and (**b**) POM from ice-algae (in purple) and snow-algae (in red) experiments with the vertical and horizontal axes (NMDS1 and NMDS2) explaining the molecular variability (grey x); the legend in the upper left details the sample labels information: type of incubation (dark /light) and time (T in days) for DOM and POM samples, and type of extracts for POM (NaOH and hot water); (**c**) and (**d**) van Krevelen diagrams with formulae according to their H/C and O/C ratios using the NMDS1 loadings ≤0.2 for ice-algae and ≥ 0.2 for snow-algae, showing how the main molecular signals in the DOM are separated; (**e**) and (**f**) van Krevelen diagrams with formulae according to their H/C and O/C ratios using the NMDS1 loadings ≤ 0.3 for glacier ice-algae and ≥ 0.3 for red snow-algae, showing how the main molecular signals in the POM are separated. Displayed in each diagram is the overall contribution (in %) of descriptive molecular categories (saturated, unsaturated aliphatics, highly unsaturated, aromatics and condensed aromatics) as well as the formulae based on their elemental composition (CHO, CHON, CHOS, CHOP) relative to the total number of formulae in the DOM or POM datasets. Note: due to insufficient particulate material in the red snow at intermediate time steps, POM could only be analyzed in the initial and final time point sample.

**
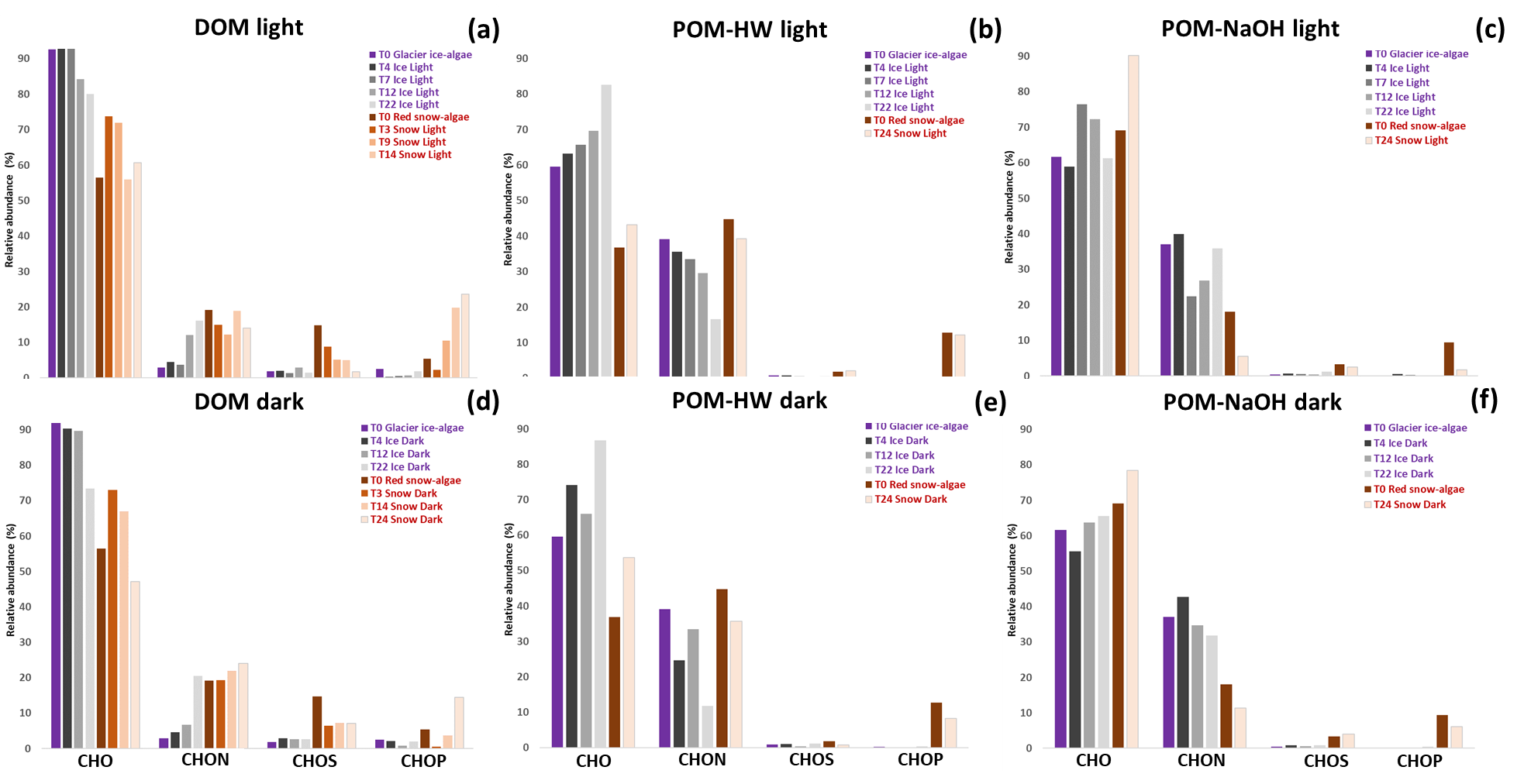
**

**Figure S5. Relative abundance of molecular formulae in the light (top row) and dark (bottom row) experiments based on their elemental composition in the DOM pools as well as the water-soluble (POM-HW) and water-insoluble (POM-NaOH) POM extracts in the molecular signal differentiated in Figure 1a and 1b for the glacier ice-algae (purple to grey toned bars) and red snow-algae (red tones) experiments**. (**a**) and **(d**) DOM light and DOM dark; (**b**) and (**e**) POM-HW light and dark and (**c**) and (**f**) POM-NaOH light and dark. Relative abundances shown in percentage relative to the total number of formulae in each sample. Note: due to insufficient particulate material in the red snow at intermediate time steps, POM could only be analyzed in the initial and final time point sample. Sulfur and phosphorus compounds were significantly more abundant in organic matter from snow-algae compared to glacier ice-algae (p < 0.05 for paired sample t-test)


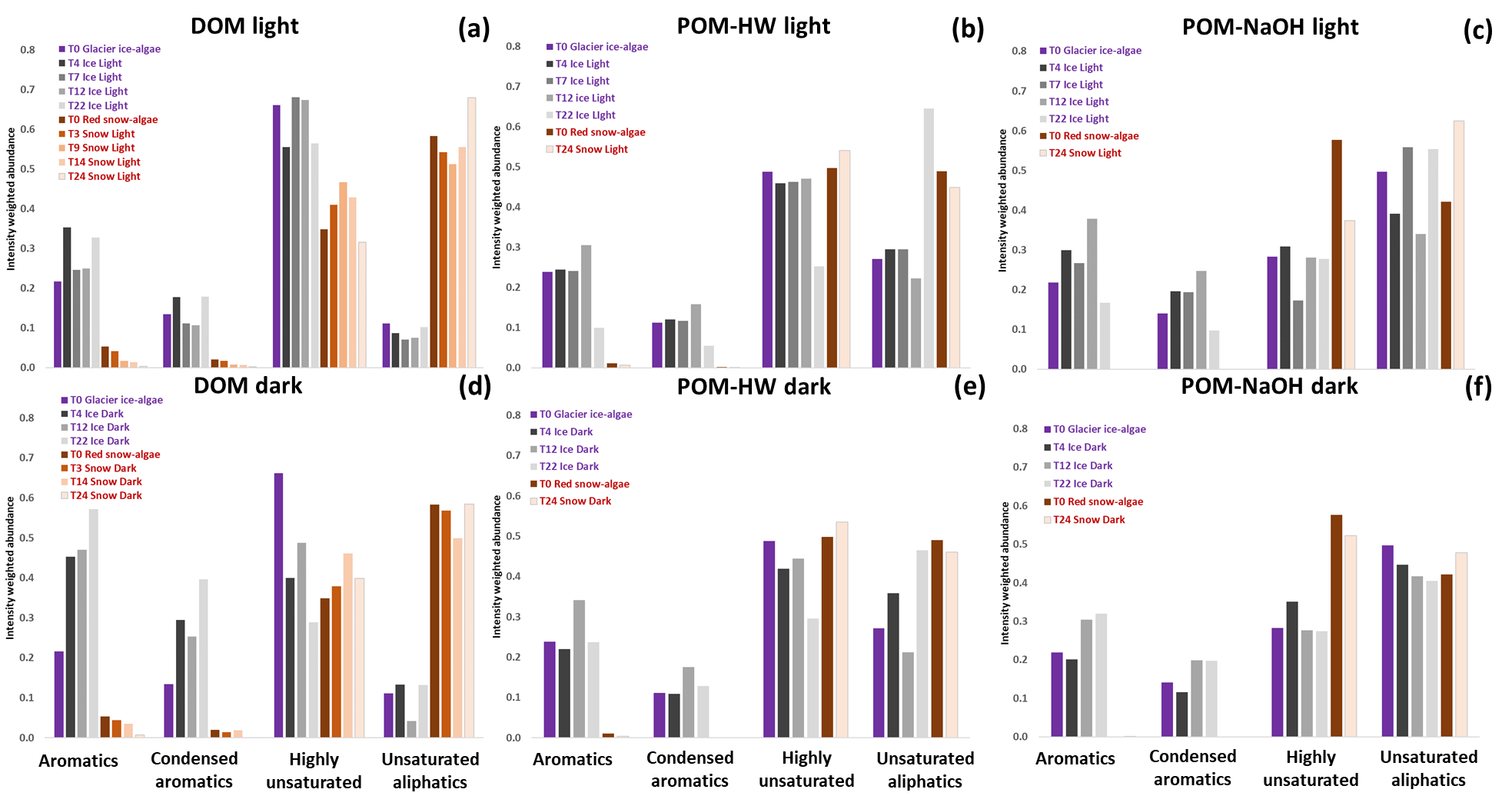


**Figure S6. Intensity weighted abundance of molecular formulae based on descriptive molecular categories in the light (top row) and dark (bottom row) experiments in the DOM pools as well as the water-soluble (POM-HW) and water-insoluble (POM-NaOH) POM extracts in the molecular signal differentiated in Figure 1a and 1b for the glacier ice-algae (purple to grey toned bars) and red snow-algae (red tones) experiments**. (**a**) and (**d**) DOM light and DOM dark, (**b**) and (**e**) POM-HW light and dark and (**c**) and (**f**) POM-NaOH light and dark. The contribution of each molecular category is based on the intensity weighted abundance of all molecular formulae within the category in each sample. Aromatics and condensed aromatics were significantly more abundant in organic matter from glacier ice-algae compared to snow-algae (p < 0.01 for paired sample t-test)

**
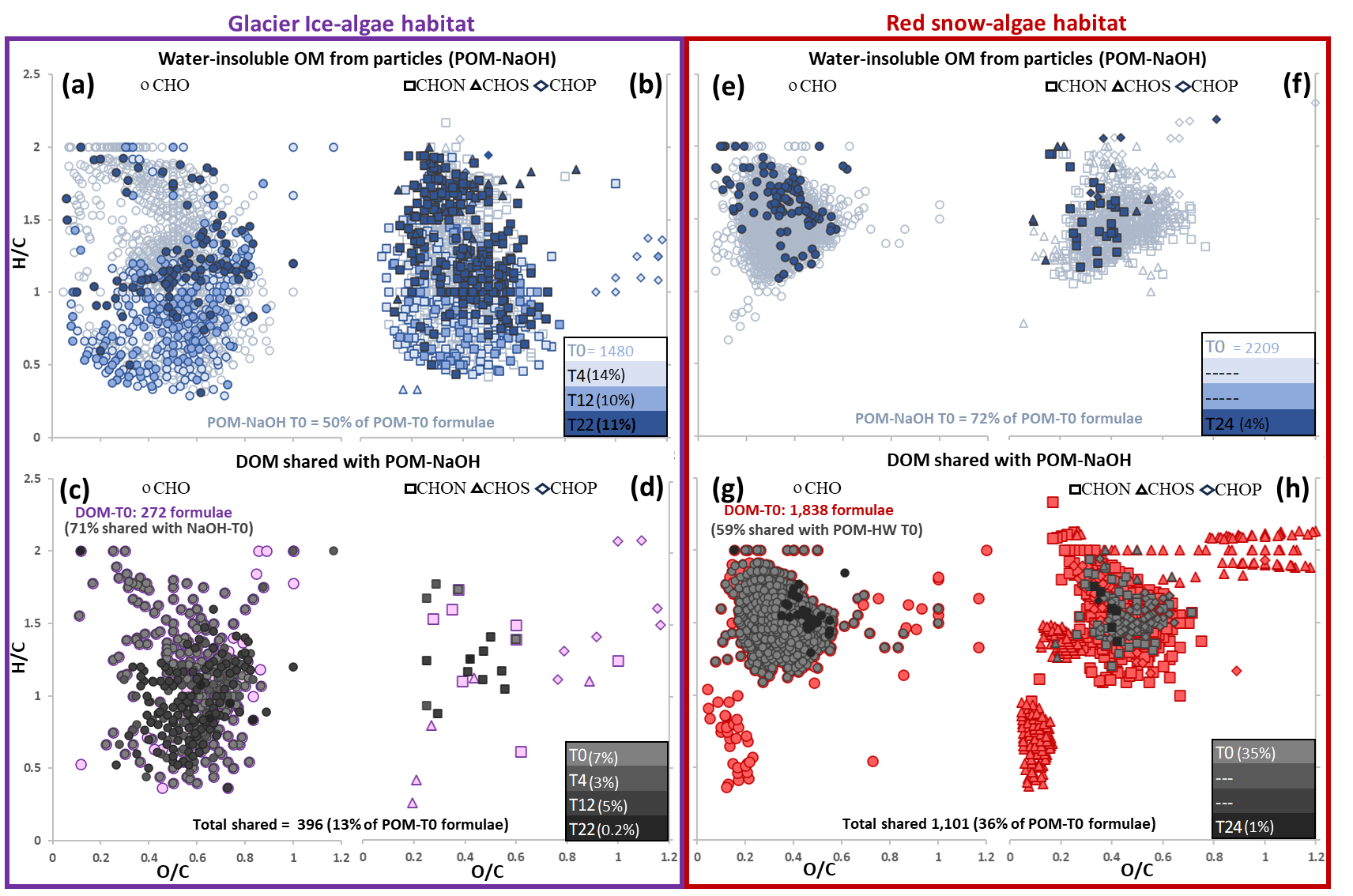
**

**Figure S7. Time resolved influence of solar radiation on the transfer of water-insoluble OM from POM to DOM in the glacier ice-algae (left panel) and red snow-algae (right panel) experiments.** van Krevelen diagrams with molecular formulae according to their H/C and O/C ratios for (**a**) and (**b)** water-insoluble ice-algae OM at T0 (empty) and over time (filled blue symbols) only under light conditions; (**c)** and (**d**) ice-algae DOM at T0 (purple) and DOM shared with water-insoluble ice-algae OM over time (dark tone symbols); (**e**) and (**f**) water-insoluble snow-algae OM at T0 (empty) and over time (filled blue symbols) only under light conditions; (**g**) and (**h**) snow-algae DOM at T0 (red) and DOM shared with water-insoluble snow-algae OM over time (dark tone symbols). Symbol shapes indicate OM without (CHO) or with heteroatoms (CHON, CHOS, and CHOP)**.** The contribution of formulae over time, indicated by the increasing dark color intensity in each panel, is expressed as percent relative to the total number of formulae in POM-T0 for glacier ice-algae (total 2,945 formulae) and red snow-algae experiments (total 3,066 formulae); for details of POM-T0 for each experiment see Tables S4 and S5. For ease of viewing DOM-T0 symbols in ice and snow are displayed in purple and red one size bigger and those uncover by dark symbols represent formulae in DOM-T0 that were not shared with the initial POM. Note: due to insufficient particulate material in the red snow at intermediate time steps, POM could only be analyzed in the initial and final time point sample.

**
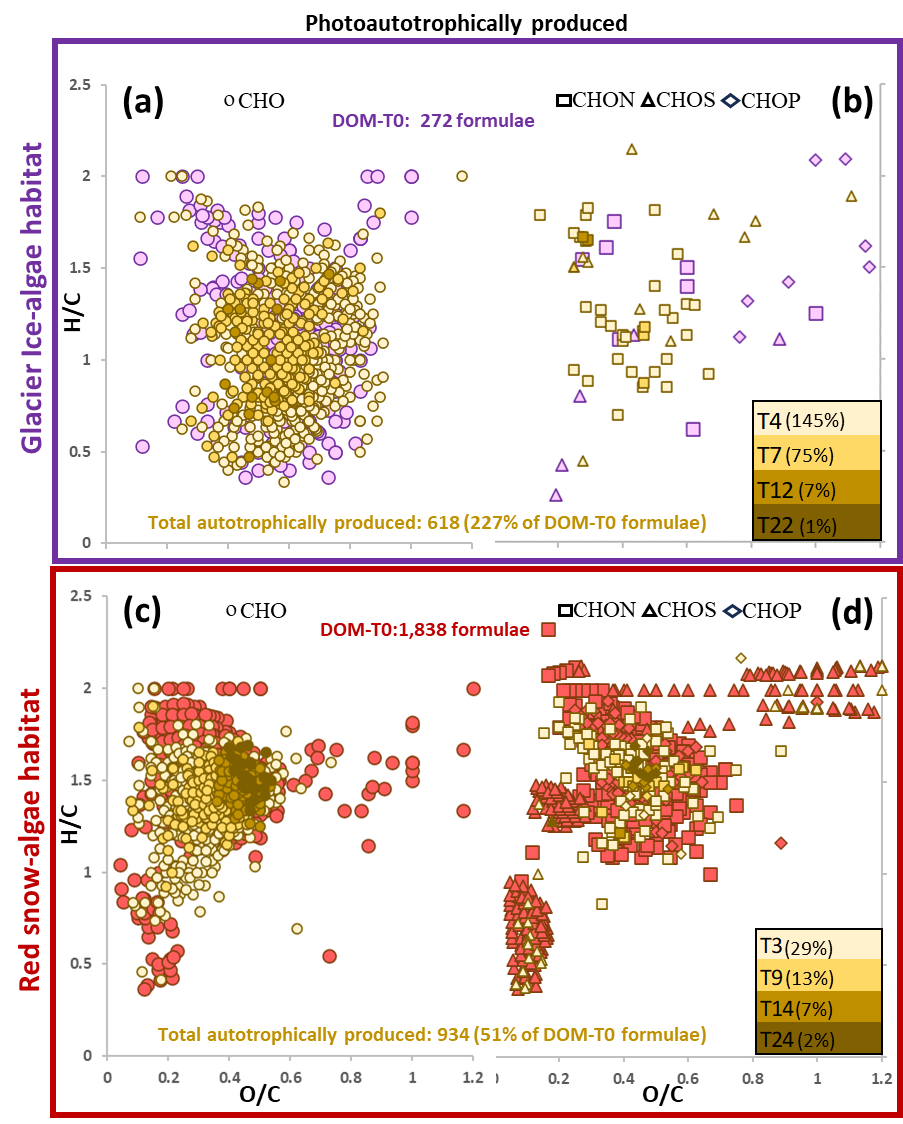
**

**Figure S8. Time resolved influence of solar radiation on the autotrophic production of DOM.** van Krevelen diagrams with molecular formulae according to their H/C and O/C ratios for (**a)** and (**b)** DOM-T0 in the glacier ice-algae experiments (purple) and molecular formulae progressively produced only under light conditions with time (in brown tones); (**c)** and (**d)** DOM-T0 in the red snow-algae experiment (dark red) and molecular formulae progressively produced only under light conditions with time (in brown tones). Symbol shapes indicate OM without (CHO) or with heteroatoms (CHON, CHOS, and CHOP). Autotrophic production was associated with a progressive increase of mass peak intensities only under light conditions. The contribution of formulae autotrophically produced over time, indicated by the increasing brown color intensity in each panel, is expressed as percent relative to the total number of formulae in DOM-T0 for glacier ice-algae and red snow-algae experiments (for details see Table S4 and S5). For ease of viewing symbols for the DOM-T0 in both panels are one size bigger than those formulae autotrophically photoproduced.

**
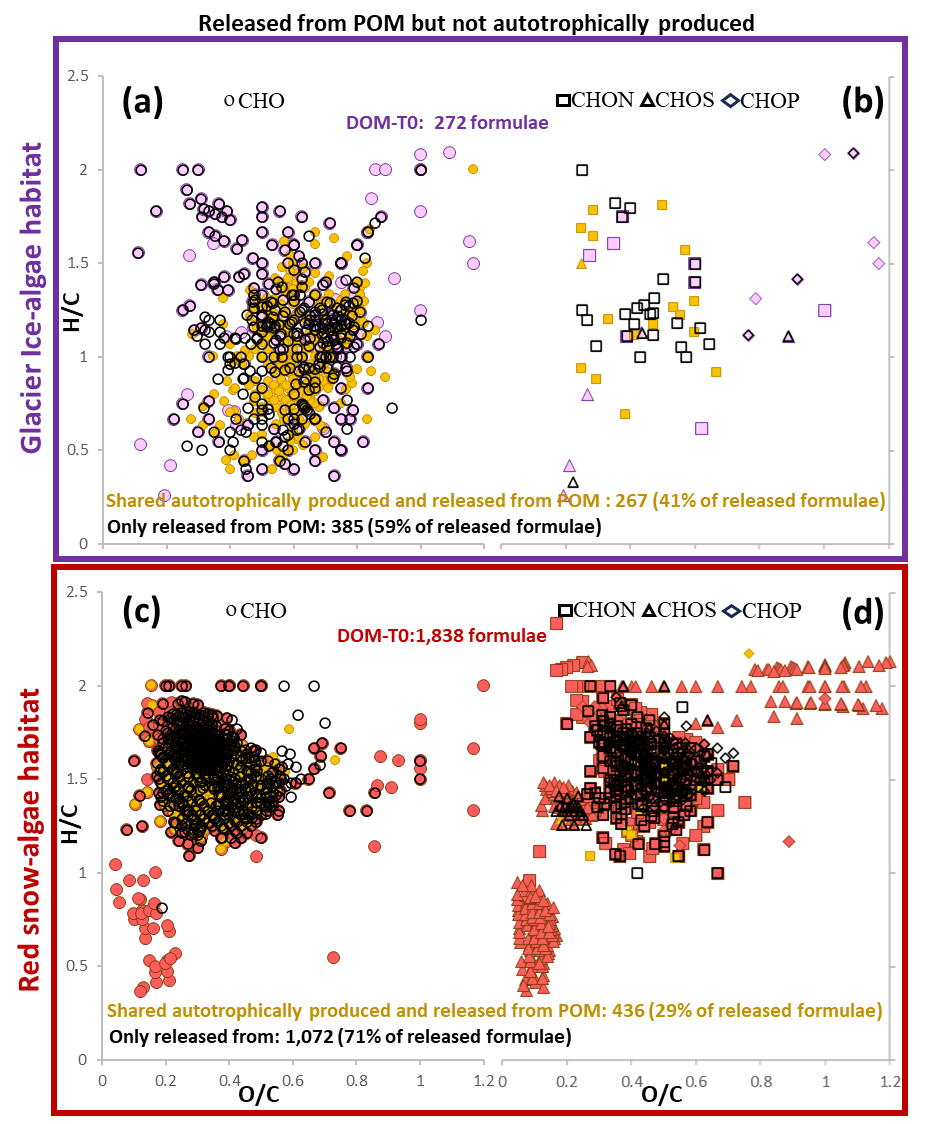
**

**Figure S9. Influence of solar radiation on the composition of DOM released from POM but not autotrophically produced.** van Krevelen diagrams with molecular formulae according to their H/C and O/C ratios for (**a**) and (**b)** DOM-T0 in the glacier ice-algae experiments (purple), shared formulae autotrophically produced and released from POM to DOM (brown) or only released from POM (black) and (**c)** and (**d)** DOM-T0 in the red snow-algae experiments (dark red), shared formulae autotrophically produced and released from POM to DOM (brown) or only released from POM (black). Symbol shapes indicate OM without (CHO) or with heteroatoms (CHON, CHOS, and CHOP). The contribution of shared formulae autotrophically produced and released from POM after solar radiation (brown color) as well as those only released from POM are expressed as percent relative to the total number of formulae released from the POM to DOM pool (for details see Table S4 and S5). Formulae released from POM to DOM but not autotrophically produced (black) represent more than half of DOM-T0 for glacier ice- and red snow-algae experiments.

**
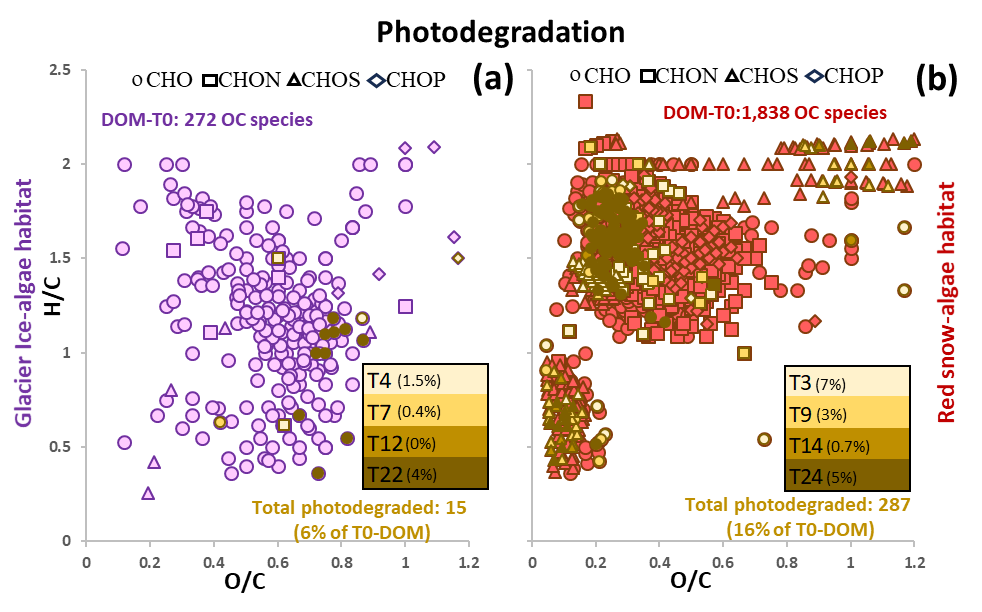
**

**Figure S10. Time resolved influence of solar radiation on the DOM degradation**. van Krevelen diagrams with molecular formulae according to their H/C and O/C ratios for (**a**) DOM-T0 in the glacier ice-algae experiments (purple) and formulae progressively degraded only under light conditions with time (in brown tones); (**b**) DOM-T0 in red snow-algae experiment (dark red) and formulae progressively degraded only under light conditions with time (in brown tones). Symbol shapes indicate CHO, CHON, CHOS and CHOP compositions. Photodegradation was associated with a progressive decrease only under light conditions of molecular formulae mass peak intensities until they reached zero or were below detection at the specified time. The contribution of formulae photodegraded over time, indicated by the increasing brown color intensity in each panel, is expressed as percent relative to the total number of formulae in DOM-T0 for glacier ice-algae and red snow-algae experiments (for details see Table S4 and S5). For ease of viewing symbols for the DOM-T0 in both panels are one size bigger than those formulae photodegraded.


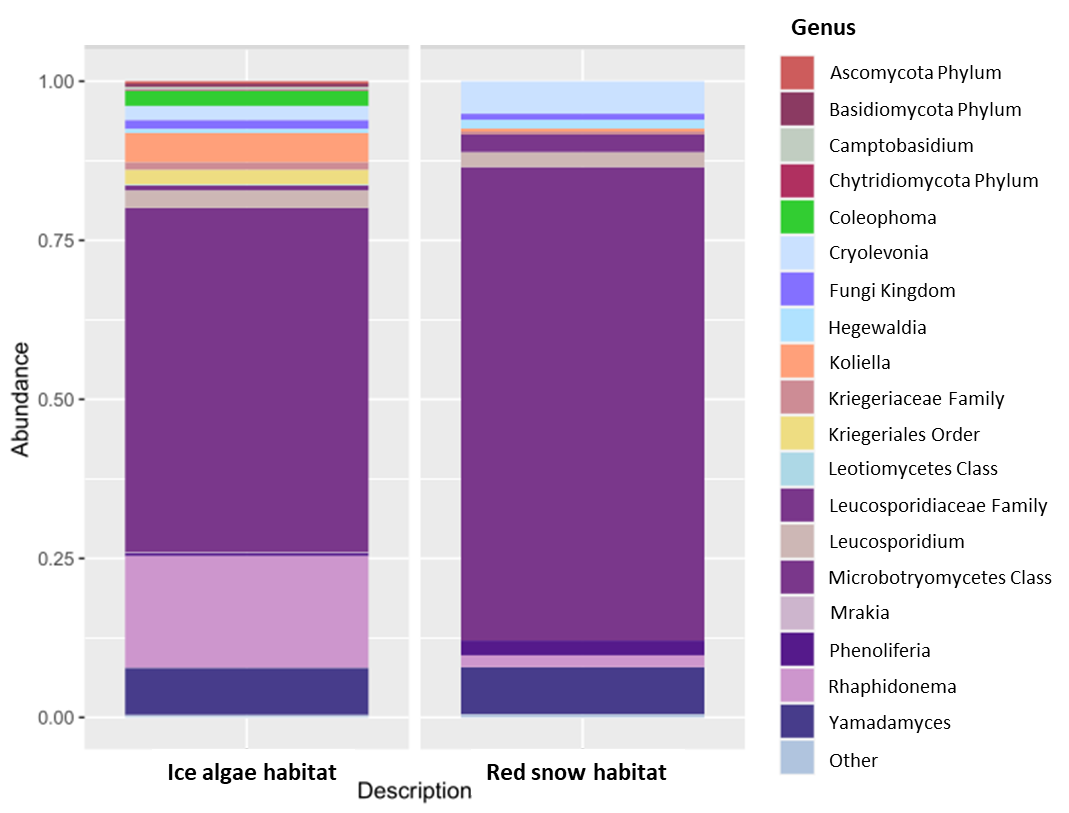


**Figure S11. Relative abundance of microbial species in the initial samples (T0_Ice and T0_Snow) based on rRNA sequencing analyses at the genus level using Internal Transcribed Spacer (ITS2) snow and ice primers (see further information in methods).**


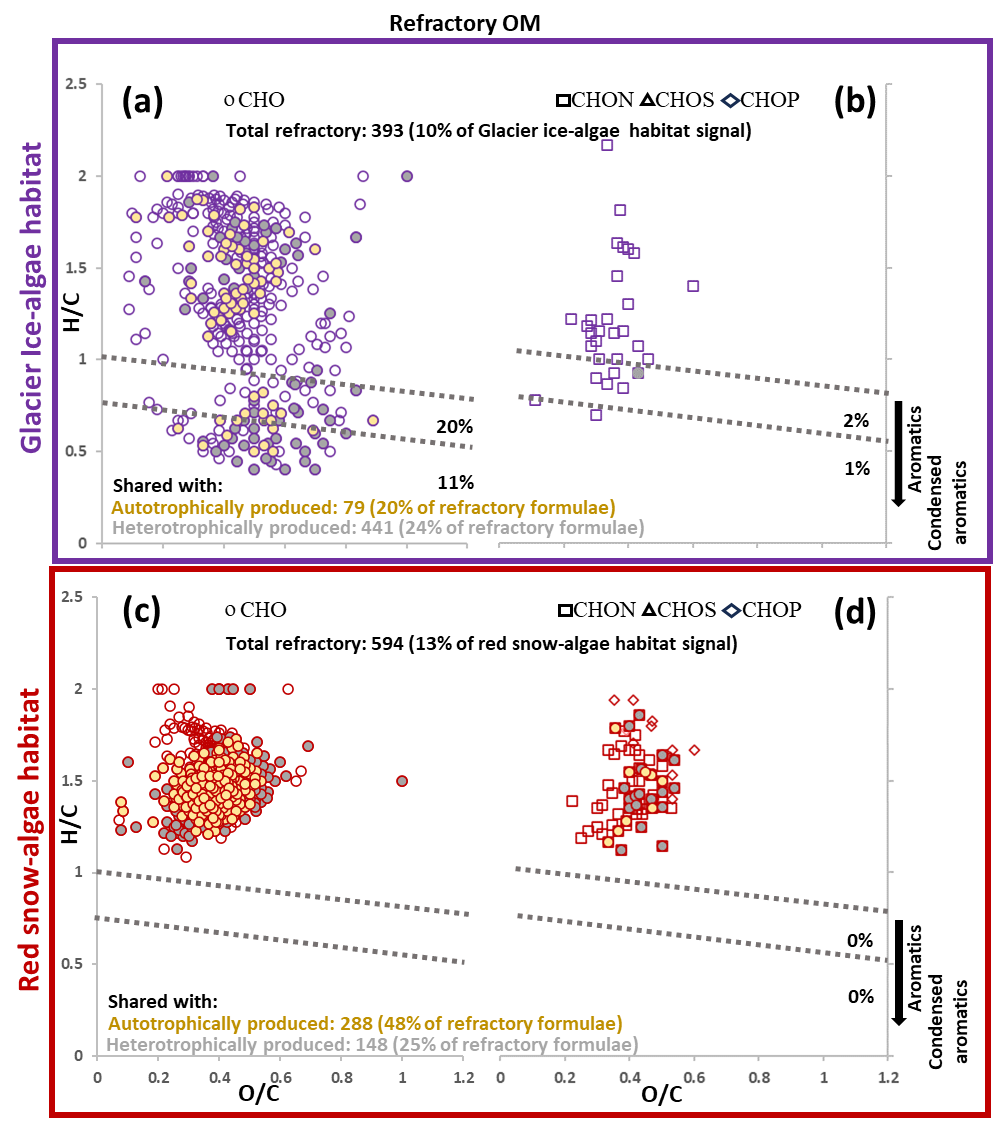


**Figure S12. Refractory OM in glacier ice- and red snow-algae dominated habitats present through the whole experiments.** van Krevelen diagrams of refractory molecular formulae that were not degraded with time and were present through the experiments according to their H/C and O/C ratios for (**a**) Glacier ice-algae (purple) and (**b)** red snow-algae (red) habitats. Refractory compounds are those that were not progressively degraded with time (by light or heterotrophic microorganisms) and were present in the DOM, POM or both pools through the whole experiment. Refractory formulae shared with those autotrophically photoproduced (yellow filled symbols) or heterotrophically produced (grey filled symbols) as well as the number of aromatics and condensed aromatics are also indicated in percentage relative to the refractory pool. Symbol shapes indicate CHO, CHON, CHOS and CHOP compositions. Total number of refractory formulae relative to the total number of formulae in each habitat (4,078 in glacier ice-algae and 4,749 in the red snow-algae from Figs. **1a** and **1b**) is also shown (for details see Table S4 and S5).
